# Effect of Brief Admission to Hospital by Self-referral for Individuals Who Self-harm and Are at Risk of Suicide: A Randomized Clinical Trial

**DOI:** 10.1001/jamanetworkopen.2019.5463

**Published:** 2019-06-07

**Authors:** Sofie Westling, Daiva Daukantaitė, Sophie I. Liljedahl, Youngha Oh, Åsa Westrin, Lena Flyckt, Marjolein Helleman

**Affiliations:** 1 Clinical Psychiatric Research Center, Department of Clinical Sciences, Lund, Psychiatry, Lund University, Region Skåne, Lund, Sweden; 2 Department of Psychology, Lund University, Lund, Sweden; 3 Department of Educational Psychology and Leadership, Texas Tech University, Lubbock; 4 Centre for Psychiatric Research, Department of Clinical Neurosciences, Karolinska Institutet, Stockholm, Sweden; 5 School of Nursing, Hanze University of Applied Sciences, Groningen, the Netherlands

## Abstract

**Importance:**

To our knowledge, there is no consensus regarding when individuals who repeatedly self-harm and are at risk of suicide should be hospitalized. To evaluate a new alternative, we examined the effects of brief admission (BA) to hospital by self-referral.

**Objectives:**

To determine the effects of BA on inpatient service use and on secondary outcomes of daily life functioning, nonsuicidal self-injuries, and attempted suicide among individuals who self-harm and are at risk of suicide.

**Design, Setting, and Participants:**

The single-masked Brief Admission Skåne Randomized Clinical Trial was conducted from September 2015 to June 2018 at 4 psychiatric health care facilities in southern Sweden. Data were collected 6 months retrospectively at baseline and at 6-month and 12-month follow-ups. Participants were randomized to either BA and treatment as usual (BA group) or treatment as usual (control group). The sample was a referral population, with the most important inclusion criteria being current episodes of self-harm and/or recurrent suicidality, at least 3 diagnostic criteria for borderline personality disorder, and hospitalization in the last 6 months.

**Interventions:**

Self-referred BA was offered for 12 months, with standard limits for duration and frequency, after the negotiation of a contract outlining the intervention.

**Main Outcomes and Measures:**

Prespecified main outcome measures were days admitted to the hospital, including voluntary admission, BA, and compulsory admission.

**Results:**

The 125 participants had a mean (SD) age of 32.0 (9.4) years, 106 (84.8%) were women, and 63 were randomized to the BA group and 62 to the control group. No significant advantage was observed in the number of days in the hospital for the BA group compared with the control group. Within-group analyses demonstrated significant decreases in both groups regarding days admitted to the hospital (BA group: χ^2^ = 22.71; *P* < .001; control group: χ^2^ = 23.01; *P* < .001) and visits to the emergency department (BA group: χ^2^ = 13.95; *P* < .001; control group: χ^2^ = 21.61; *P* < .001), but only the BA group showed a reduction in days with compulsory admission (χ^2^ = 7.67; *P* = .02) and nonsuicidal self-injuries (χ^2^ = 6.13; *P* = .047). The BA group showed significantly greater improvements in the mobility domain of daily life functioning (*z* = −2.39; *P* = .02) and significant within-group improvements in 3 other domains (cognition: *F* = 9.02; *P* < .001; domestic responsibilities: *F* = 3.23; *P* = .049; and participation: *F* = 3.79; *P* = .03).

**Conclusions and Relevance:**

Brief admission appears no more efficacious in reducing use of inpatient services than usual care for individuals who self-harm and are at risk of suicide. Future studies should explore other possible beneficial effects.

**Trial Registration:**

ClinicalTrials.gov identifier: NCT02985047

## Key Points

**Question:**

Is self-referred brief admission more effective than treatment as usual in reducing the use of inpatient services for individuals who self-harm and are at risk of suicide?

**Findings:**

In this randomized clinical trial of 125 adults in Sweden, the brief admission group did not show reduced inpatient service use compared with the control group. Both groups showed significant decreases in days admitted to the hospital and in emergency department visits, but only the brief admission group showed a significant decrease in duration of compulsory admission.

**Meaning:**

Brief admission does not seem to be more effective than treatment as usual in reducing the use of inpatient services.

## Teaser

This randomized clinical trial determines the effects of brief admission to hospital by self-referral vs usual treatment on inpatient service use, daily life functioning, nonsuicidal self-injuries, and attempted suicide among Swedish adults who self-harm and are at risk of suicide.

## Introduction

Brief admission (BA) by self-referral is an intervention allowing individuals to hospitalize themselves. Duration (ie, length of stay) and frequency (ie, number of admissions per month) are limited. Preliminary small and qualitative studies have yielded promising results. In a Dutch mixed-methods study,^1^ 11 participants with histories of long hospitalization were offered access to BA by self-referral with 6-month follow-up. The results demonstrated that inpatient service use decreased over time, albeit nonsignificantly, and participants were content with the intervention. Similar initiatives were conducted in Norway. A meta-analysis of 6 qualitative and small quantitative studies using Norwegian data^2^ showed promising results; however, this was rather low-grade evidence. Quantitative studies revealed a large reduction in inpatient care use among individuals with access to patient-controlled admissions.^2^ Qualitative studies suggest that such admissions increased individuals’ autonomy, responsibility, and self-confidence in daily life.^2^

A 2018 study^3^ and a 2019 study^4^ made greater headway. A Norwegian randomized clinical trial^3^ (RCT) examined 54 participants with schizophrenia and bipolar disorder, randomizing 26 to self-referral for inpatient treatment.^3^ The primary outcomes were number of days as inpatients, number of admissions, outpatient contacts, and coercion. While the intervention group had somewhat more hospital admissions (including self-referral admissions), no significant differences were found between the groups in any other outcomes. A national Danish matched prospective cohort study^4^ investigated whether patient-controlled admissions could reduce inpatient service use, compulsory measures, medication, and self-harm. They reported no significant differences between the intervention arm and the treatment-as-usual (TAU) control arm in compulsory measures and self-harm. However, the control group used fewer inpatient services and less medication.

Thus, previous research is inconclusive. Despite increasing interest in hospitalization by self-referral, there appear to be few rigorous studies on its effectiveness. Further, to our knowledge, no previous studies used fully standardized interventions or reported measures for ensuring treatment fidelity. Participants in prior studies were mainly individuals with severe psychotic and bipolar disorders and extensive prior use of inpatient care. Another possible target group for the intervention is people who recurrently self-harm and are at risk of suicide, as general admissions might be harmful for this group.^5,6,7^ Furthermore, admissions remain frequent and long because of these individuals’ high risk of suicide and self-harm-related sequelae.^5,8^

In this RCT, the primary objective was to evaluate the effects of a standardized version of BA by self-referral on inpatient and compulsory care among individuals who self-harm and are at risk of suicide. The secondary objective was to examine whether BA by self-referral increases individuals’ daily functioning and reduces their frequency of self-harm.

## Methods

The Brief Admission Skåne Randomized Clinical Trial was a single-masked RCT conducted throughout the region of Skåne, Sweden (population, 1.3 million). Participants were recruited from all 4 psychiatric inpatient clinics in the region. This study followed the Consolidated Standards of Reporting Trials (CONSORT) reporting guideline^9^ and was approved by the Regional Ethics board at Lund University. All participants gave written informed consent accordingly. No incentives were offered. The trial protocol (Supplement 1) has been published previously.^10^

### Participant Selection, Recruitment, and Enrollment

All practitioners working at the clinics were informed of the inclusion and exclusion criteria. The inclusion criteria were (1) current episodes of self-harm and/or recurrent suicidal behavior, (2) 3 or more diagnostic criteria for borderline personality disorder,^11^ (3) 7 or more days of hospital admission or presenting to an emergency department 3 or more times in the last 6 months, and (4) age 18 to 60 years. The exclusion criteria were (1) absence of regular contact with an outpatient psychiatric clinic, (2) unstable housing (eg, homeless, imprisoned), and (3) a nonpsychiatric disorder that significantly affects the inclusion criteria (eg, self-harm only occurs during hypoglycemia among individuals with diabetes). Overall, 129 individuals gave informed consent and were assessed for eligibility; 4 did not meet the inclusion criteria (2 did not have 3 or more diagnostic criteria for borderline personality disorder, 1 did not engage in self-harm, and 1 did not have regular contact with the outpatient clinic) (Figure). Thus, 125 participants were recruited. The number of participants was determined via a prespecified power analysis.^12,13^ Following the pilot phase, interim analyses were conducted to evaluate the fidelity measures and make minor changes to the language of the participant documents (ie, the BA contract and intervention-specific questionnaires). No changes were made to the content; the changes focused on clarifying the language and structure to participants. Data from the pilot phase were included in the study because none of these changes would affect the outcome measures.

**Figure.**
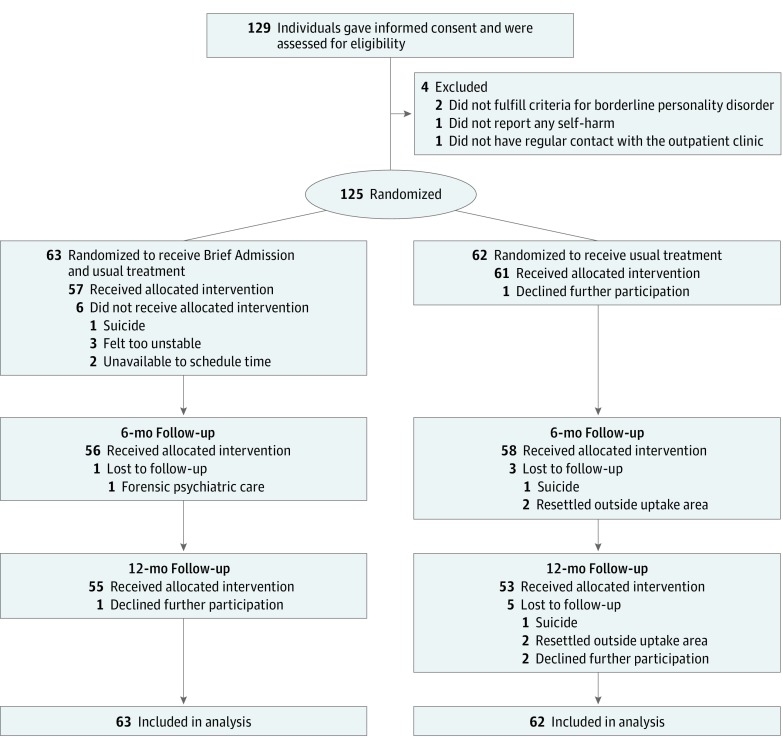
CONSORT Diagram of Participant Flow During the Study^9^

### Randomization

Participants were randomized at the individual level (allocation ratio 1:1) to either BA and TAU (BA group) or TAU (control group). Block randomization was applied, using tables of random numbers in blocks of 4, stratified according to the inpatient unit where BA was provided. One of us (D.D.) generated the allocation sequence, and a research nurse prepared consecutively numbered randomization envelopes containing information on allocation. Recruitment staff were masked to participants’ randomization status. One of us (S.W.) enrolled participants and gave eligible participants their randomization envelope, which they opened and signed. Participants learned their treatment assignment after eligibility assessment.

### Intervention

Helleman et al^14,15^ extracted the core BA components. These components were standardized by using an education manual, a set implementation process, a fidelity measure, and evaluation questionnaires.^16^ Before beginning the intervention, participants in the BA group negotiated a contract, which states the parameters of the admission as well as its specific components. The individual commits to goals for BA, preferred approach from staff, and stress-reducing activities. The contract states responsibilities during BA, such as the need to bring and administer medication, not to harm oneself or others, not to be under the influence of alcohol or illegal drugs, and not to be aggressive. Table 1 outlines the core components and the relevant comparisons with general hospital admissions.

**Table 1.** Core Components of BA by Self-referral^10^ Compared With General Admission

Component	BA	General admission
Contract	The contract states the parameters of the admission as well as its specific components, in which individuals commit to goals for BA, preferred approach from staff, and stress-reducing activities. Responsibilities during BA are stated, such as the need to bring and administer medication, not to harm themselves or others, not to be under the influence of alcohol or illegal drugs, and not to be aggressive.	NA
Negotiation	The contract is discussed, written, and signed by individuals seeking BA, their outpatient clinician, and a nurses’ aide or nurse from the BA ward. The individuals seeking BA receive complete information about the intervention. All individualized parts of the contract are discussed and documented.	NA
Approach	The approach is explicit and should be characterized by warmth, enthusiasm, acceptance, genuineness, openness, validation of current difficulties, and praise of the individuals for managing symptoms by seeking BA.	NA
Receiving admission	Individuals decide when to be admitted with a maximum duration and frequency (ie, 3 nights in a row, 3 times per month). Nurses’ aide or nurse has an admission conversation.	Individuals seek admission but physician makes the decision. Physician and nurse have admission conversations.
During admission	Individuals can have 1-2 daily conversations with ward staff and participate in activities at the ward but not engage in any contact with the ward physician, not change treatment, and not receive medication from the ward. Individuals are responsible for their safety.	Conversations for planning treatment takes place among clinicians across disciplines. Medication is provided by the ward. The ward is responsible for safety.
Discharge	Individuals are discharged by a nurses’ aide or a nurse. Admission longer than 3 nights is not negotiable.	Individuals are discharged after decision by a senior physician. Negotiable length of admission.
Premature discharge	Not following the commitments in the contract (eg, engaging in self-harm) results in premature discharge alongside a discussion of what went wrong for the purpose of future learning.	Self-harm or suicidal behavior usually results in prolonged admissions.

Abbreviations: BA, brief admission; NA, not applicable.

### Treatment Fidelity

The best practices from the National Institutes of Health Behavior Change Consortium^17^ were adhered to as closely as possible for all 5 domains of treatment fidelity: study design, clinician training, treatment delivery, treatment receipt, and treatment enactment.^18,19^ For more detailed information, see eTable 1 in Supplement 2.

### Assessments

A diagnostic psychiatric assessment was performed by one of us (S.W.) before randomization with the Mini*-*International Neuropsychiatric Interview^20^ and Structured Clinical Interview for *Diagnostic and Statistical Manual of Mental Disorders* (Fourth Edition) (*DSM*-*IV*) Axis II disorders.^21^ Information on attention-deficit/hyperactivity disorder and autism was retrieved from medical records.

### Outcome Measures

The prespecified primary outcome measures were days admitted to hospital, including voluntary admission, BA by self-referral, and compulsory admission. These data were retrieved from medical records 6 months retrospectively at study inclusion (T1), at 6-month follow-up (T2), and at 12-month follow-up (T3).

The secondary outcome measures were (1) frequency of compulsory measures (eg, restraints, forced treatment, and shielding), (2) scores on the World Health Organization Disability Assessment Schedule II^22^ (WHODAS II), and (3) scores on the 5 Self-harm Behavior Groupings Measure.^23^ The WHODAS II is a 36-item self-rating questionnaire assessing functioning in 6 domains: cognition (6 items; understanding and communicating; eg, “concentrating on doing something for ten minutes”; Cronbach α for both groups at T1, 0.83), mobility (5 items; moving and getting around; eg, “getting out of your home”; Cronbach α for both groups at T1, 0.83), self-care (4 items; hygiene, dressing, eating, and staying alone; eg, “washing your whole body”; Cronbach α for both groups at T1, 0.56), getting along (5 items; interacting with others; eg, “maintaining a friendship”; Cronbach α for both groups at T1, 0.72), life activities (4 items; domestic responsibilities only; eg, “taking care of your household responsibilities”; Cronbach α for both groups at T1, 0.95), and participation (8 items; joining in community activities; eg, “how much of a problem did you have living with dignity because of the attitudes and actions of others?”; Cronbach α for both groups at T1, 0.84).^22^ The items are scored as follows: 1, no difficulty; 2, mild difficulty; 3, moderate difficulty; 4, severe difficulty; and 5, extreme difficulty/cannot do. Item scores were then recoded and summed in each domain (ranging from 0 [best] to 100 [worst]) using complex scoring. An SPSS algorithm available from the World Health Organization was used.^22^ The 5 Self-harm Behavior Groupings Measure was developed to assess and grade self-harming behaviors in terms of directness (direct or indirect self-harm), severity and lethality, and suicidal intent. For this study, data on nonsuicidal self-injuries^11^ (NSSIs) and attempted suicide were retrieved. All self-report assessments were performed at T1 and both follow-ups.

### Adverse Events

All participants exhibited recurrent self-harm; thus, self-harm was only logged as an adverse event if it occurred during BA. Self-harm acts during BA were considered unfavorable or unintended events that resulted in a discharge from the current BA but did not affect the contract. When behaviors increased stress or risk for other patients at the ward, the BA contract was paused until the individual reported being able to avoid such behaviors in the future. Escalating self-harm, suicidality, or endangerment of others in the ward among more than 3 individuals in the BA group were the stopping guidelines for the RCT.

### Statistical Analysis

Latent growth curve modeling (LGCM)^24,25,26^ was performed to explore the latent growth trajectories for continuous outcomes. Zero-inflated Poisson (ZIP) models were used for count variables with excessive 0 outcomes. The impact of the time-invariant covariate group (0, control; 1, BA) on the initial intercept (or counts for count variables) and the rate of change in the slope (or counts) over time was the focus of inference. A significant result indicated that the groups had significantly different developmental patterns over the time points.

Of the 3 primary outcomes, days with compulsory admission and number of visits to the emergency department were fitted with conditional ZIP growth curve models, with group (0, control; 1, BA) as a time-invariant covariate. Days admitted to hospital had almost no 0 counts (less than 9% at each time point) and therefore was treated as a continuous variable (vs a count variable).

Before conducting the analysis of the secondary outcomes, missing data were handled by an iterative Markov chain Monte Carlo imputation technique.^27^ The attrition is described in the Results section. The number of imputations (100) was chosen with reference to Bodner^28^ and White et al^29^ using 4 auxiliary variables (age, educational level, borderline personality disorder, and sex) to help to predict the missing values.^30^ These analyses were conducted using Mplus version 8 (Muthén and Muthén).^31^

The number of compulsory measures had an extreme number of 0 counts (88% at T1, 87.2% at T2, and 80.8% at T3). Therefore, it was dichotomized.

To evaluate group and sex differences at T1, *t* tests were performed for normally distributed continuous variables, Mann-Whitney *U* tests for nonnormally distributed continuous variables, and Pearson χ^2^ tests for categorical variables. Further, within-group differences were analyzed via hierarchical linear modeling with time (coded as 0, 1, and 2) as a fixed effect for normally distributed continuous variables and Friedman test for nonnormally distributed variables. Cohen *d* values were calculated to evaluate the effect sizes of the within-group effects from T1 to T3 using the mean differences (for continuous variables) divided by the full-sample SD at T1 and by taking the correlation between the 2 repeated measures into account, as suggested by Morris and DeShon.^32^ *P *values less than .05 were considered significant, and all tests were 2-tailed. The within-group analyses were exploratory and performed using IBM SPSS Statistics version 25 (IBM Corp).^33^

## Results

From September 1, 2015, to June 30, 2017, 125 individuals were included in the study and evaluated through June 30, 2018. Mean (SD) age was 32.0 (9.4) years, and 106 (84.8%) were women. Of the 125 participants, 63 were randomized to the BA group and 62 to the control group. Overall, 57 BA group members (90.5%) and 61 control group members (98.4%) received the allocated condition (Figure). During follow-up, 2 further individuals in the BA group and 8 individuals in the control group dropped out of the study, resulting in a final sample of 108 individuals (55 individuals in the BA group and 53 individuals in the control group). The completion rate was 86.4%. Attrition analyses were conducted, and no significant differences were found between the groups on baseline scores for any variable. The reasons for discontinuation and number of adverse events were similar in both groups.

Baseline demographic and clinical variables by treatment group are reported in Table 2. No significant differences were observed at T1 between the groups other than that the BA group contained more individuals with anxiety disorders and more days of compulsory admission. No significant sex differences were observed in any of the outcomes at T1, except for NSSI (*U* = 393; *z* = −1.99; *P* = .047).

**Table 2.** Baseline Sociodemographic and Clinical Characteristics by Randomization Status

Characteristic	No. (%)			Test Statistic	*P* Value^a^
	BA Group (n = 62)	Control Group (n = 63)	Total (N = 125)		
Age, mean (SD), y	30.9 (8.8)	33.1 (9.9)	32.0 (9.4)	*t* = −1.30	.20
Women	56 (90.3)	50 (79.4)	106 (84.8)	χ^2^ = 2.91	.09
Education					
≤Elementary school	23 (37.1)	20 (31.7)	43 (34.4)	χ^2^ = 2.62	.27
High school degree	24 (38.7)	33 (52.4)	57 (45.6)		
≥Bachelor’s degree	15 (24.2)	10 (15.9)	25 (20.0)		
Living alone	33 (53.2)	32 (50.8)	65 (52.0)	χ^2^ = 0.07	.79
Living with partner	25 (40.3)	24 (38.1)	49 (39.2)	χ^2^ = 0.07	.80
Accommodation with access to staff					
During parts of the day	3 (4.8)	0	3 (2.4)	χ^2^ < 0.01	>.99
Throughout the day	5 (8.1)	7 (11.1)	12 (9.6)	χ^2^ = 0.33	.56
Child at home	15 (24.2)	17 (27.0)	32 (25.6)	χ^2^ = 0.13	.72
Mental illness symptoms					
Suicidal ideation in last mo	61 (96.8)	60 (96.8)	121 (96.8)	χ^2^ < 0.01	>.99
Suicidal behavior in last y	50 (79.4)	50 (80.6)	100 (80.0)	χ^2^ = 0.03	.86
Mental disorders					
Anxiety	31 (50.0)	20 (31.7)	51 (40.8)	χ^2^ = 4.31	.04
Bipolar and related	21 (33.9)	21 (33.3)	42 (33.6)	χ^2^ < 0.01	>.99
Borderline personality	33 (53.2)	40 (63.4)	73 (58.4)	χ^2^ = 1.36	.24
Eating	16 (25.8)	12 (19.0)	28 (22.4)	χ^2^ = 0.82	.37
Depressive disorder	46 (74.2)	42 (66.7)	88 (70.4)	χ^2^ = 0.85	.36
Obsessive-compulsive	11 (17.7)	11 (17.5)	22 (17.6)	χ^2^ < 0.01	>.99
Personality (borderline excluded)	10 (16.1)	14 (22.2)	24 (19.2)	χ^2^ = 0.75	.39
Posttraumatic stress	29 (46.8)	27 (42.9)	56 (44.8)	χ^2^ = 0.19	.66
Psychotic	3 (4.8)	3 (4.8)	6 (4.8)	χ^2^ < 0.01	>.99
Substance-related	23 (37.1)	32 (50.8)	55 (44.0)	χ^2^ = 2.38	.12
No. of psychotropic medicaments, mean (SD)	5.7 (2.0)	5.1 (1.9)	5.4 (2.0)	*t*_110_ = 1.67	.10
No. of NSSIs in last 2 wk, mean (SD) [median]	6.8 (7.6) [5.0]	5.4 (8.2) [3.5]	6.2 (7.9) [4.0]	*U* = 535.00	.26
No. of suicide attempts in last 2 wk, mean (SD) [median]	0.2 (0.7) [0]	0.4 (0.9) [0]	0.3 (0.8) [0]	*U* = 878.00	.13
Admitted to hospital, mean (SD) [median], d	58.9 (48.2) [40.0]	49.4 (38.8) [38.0]	54.2 (43.9) [39.0]	*U* = 1797.00	.44
Compulsory admission to hospital, mean (SD) [median], d	15.4 (29.3) [0]	9.7 (30.7) [0]	12.6 (30.0) [0]	*U* = 1576.50	.03
No. of compulsory measures, eg, restraint, forced treatment, mean (SD) [median]	0.6 (1.8) [0]	0.2 (0.7) [0]	0.4 (1.4) [0]	*U* = 1790.50	.16
Visits to emergency department, mean (SD) [median]	6.0 (6.2) [3.0]	5.0 (5.5) [3.5]	5.5 (5.9) [3.0]	*U* = 1721.50	.25
WHODAS II domains, mean (SD)					
Cognition	53.8 (20.6)	54.5 (21.5)	54.1 (21.0)	*t*_108_ = −0.17	.87
Mobility	45.7 (26.3)	41.2 (25.7)	43.5 (26.0)	*t*_104_ = 0.89	.38
Self-care	37.0 (21.0)	41.3 (23.6)	39.2 (22.3)	*t*_105_ = −0.99	.32
Getting along	56.7 (25.2)	60.8 (25.9)	58.6 (25.5)	*t*_102_ = −0.81	.42
Domestic responsibilities	66.4 (28.4)	66.6 (30.6)	66.5 (29.4)	*t*_106_ = −0.04	.97
Participation	61.4 (19.6)	60.8 (21.5)	61.1 (20.5)	*t*_95_ = 0.15	.88

Abbreviations: BA, brief admission; NSSI, nonsuicidal self-injury; WHODAS II, World Health Organization Disability Assessment Schedule II.

^a^ Significance test results are from *t* tests for normally distributed continuous variables (*t* statistic), Mann-Whitney *U* tests for nonnormally distributed continuous variables (*U *statistic), and Pearson χ^2^ tests for categorical variables (χ^2^ statistic).

### Primary Outcomes

Table 3 provides the descriptive statistics, the between-group differences, and the within-group differences in the primary outcome variables at all time points. The overall fit statistics for the LGCM and ZIP curve growth models are presented in eTable 2 and eTable 3 in Supplement 2.

**Table 3.** Within-Group Differences for Primary Outcome Variables, NSSI, and Suicide Attempts

Variable	BA Group, Mean (SD) [Median]					Control Group, Mean (SD) [Median]					LGCM With Group as Covariate	
	T1	T2	T3	Within-Group Difference^a^		T1	T2	T3	Within-Group Difference^a^		Estimate (SE) for Slope	*P* Value
				χ^2^	*P* Value				χ^2^	*P* Value		
Total days admitted to hospital^b^^,^^c^	59.95 (49.88) [41.00]^d^^,^^e^	45.45 (50.08) [27.00]^d^^,^^f^	30.14 (44.98) [7.50]^e^^,^^f^	22.71	<.001	51.55 (39.53) [39.00]^d^^,^^e^	40.56 (48.21) [21.00]^d^^,^^f^	29.44 (40.61) [10.00]^e^^,^^f^	23.01	<.001	−4.06 (4.19)	.33
Days with general admission^b^	59.95 (49.88) [41.00]^d^^,^^e^	35.86 (45.81) [15.50]^d^^,^^f^	24.04 (44.02) [4.00]^e^^,^^f^	35.38	<.001	51.55 (39.53) [39.00]^d^^,^^e^	40.56 (48.21) [21]^d^^,^^f^	29.44(40.61) [10.00]^e^^,^^f^	23.01	<.001	−6.06 (4.24)	.15
Days with compulsory admission^b^	15.43 (29.32) [0]^d^^,^^e^	8.12 (19.46) [0]^d^	7.34 (20.35) [0]^e^	7.67	.02	9.66 (30.67) [0]	9.80 (34.88) [0]	5.55 (15.71) [0]	3.39	.18	−0.33 (0.31)	.29
Visits to an emergency department^b^	4.36 (4.93) [3.00]^e^	3.91 (6.24) [1.50]	3.52 (6.48) [1.00]^e^	13.95	<.001	6.09 (6.38) [4.00]^d^^,^^e^	4.64 (6.59) [3.00]^d^^,^^f^	2.95 (3.52) [2.00]^e^^,^^f^	21.61	<.001	0.19 (0.12)	.12
NSSI^g^	6.84 (7.62) [5.00]^e^	4.86 (8.43) [0]	4.41 (6.58) [1.00]^e^	6.13	.047	5.44 (8.18) [3.50]	5.15 (7.00) [2.00]	4.44 (6.11) [0.50]	1.07	.59	−0.41 (0.24)	.09
Suicide attempts^g^	0.23 (0.68) [0]	0.30 (0.72) [0]^d^	0.03 (0.18) [0]^d^	6.75	.03	0.40 (0.90) [0]	0.19 (0.75) [0]	0.03 [0] (0.19)	1.00	.61	NA^h^	NA^h^

Abbreviations: BA, brief admission; LGCM, latent growth curve modeling; NA, not applicable; NSSI, nonsuicidal self-injury; T1, baseline; T2, 6-month follow-up; T3, 12-month follow-up.

^a^ Friedman test was used to evaluate the within-group differences.

^b^ Measured using the 6-month time period.

^c^ Total days admitted to hospital includes days with general admission, compulsory admission, and BA.

^d^ Signficant difference found between mean and median from T1 to T2.

^e^ Signficant difference found between mean and median from T1 to T3.

^f^ Signficant difference found between mean and median from T2 to T3.

^g^ Measured for 2 weeks.

^h^ Only 20 participants in the BA group and 29 participants in the control groups supplied information on suicide attempts at T1, T2, and T3. The sample was therefore considered too small to perform LGCM.

Between-group analyses showed no significant differences between the groups in the number of days admitted to the hospital (LGCM, −4.06; *z* = −0.97; *P* = .33). Within-group analyses showed significant decreases in both groups over the 3 time points (BA group: χ^2^ = 22.71; *P* < .001; control group: χ^2^ = 23.01; *P* < .001) (Table 3). Approximately 20% of total admission days for the BA group were for BA by self-referral at both T2 and T3 (eFigure 1 in Supplement 2).

The BA group did not show a significantly greater decrease in the number of days with compulsory admission than the control group (LGCM, −0.33; *z* = −1.06; *P* = .29) (Table 3). However, significant within-group differences were found in the BA group (χ^2^ = 7.67; *P* = .02) but not the control group (χ^2^ = 3.39; *P* = .18).

Regarding the number of visits to the emergency unit, the BA group did not show a significantly greater decrease in the number of visits than the control group (LGCM, 0.19; *z* = 1.54; *P* = .12) (Table 3). Significant within-group decreases were found in both groups (BA group: χ^2^ = 13.95; *P* < .001; control group: χ^2^ = 21.61; *P* < .001).

Different compulsory measures (eg, restraints, forced treatment, and shielding) were applied for 10 individuals in the BA group (15.9%) and 5 individuals in the control group (8.1%) at T1. No significant decrease between the groups and no significant differences within the groups were found at any time point.

### Secondary Outcomes

The effects of the intervention on the 6 domains of the WHODAS II were also evaluated. For the life activities domain, only domestic responsibilities were evaluated because most participants (approximately two-thirds of both groups) could not reply to the items regarding work and school, mainly owing to current sick leave. The overall fit statistics for the LGCM are presented in eTable 4 in Supplement 2.

As shown in Table 4 and eFigure 2 in Supplement 2, when the LGCM models were run with group as a covariate, a significantly greater decrease was observed in the growth trajectory of mobility in the BA group (LGCM, −5.59; *z* = −2.39; *P* = .02) compared with the control group. Further, significant within-group differences were found in 4 domains in the BA group (cognition: *F*_2.0,48.5_ = 9.02; *P* < .001; mobility: *F*_2.0,45.4_ = 11.42; *P* < .001; domestic responsibilities: *F*_2.0,43.6_ = 3.23; *P* = .049; and participation: *F*_2.0,40.4_ = 3.79; *P* = .03), but no significant within-group differences were found for the control group.

**Table 4.** Within-Group Differences for Secondary Outcome Variables by Treatment Group

WHODAS II Measure	BA Group, Mean (SD)						Control Group, Mean (SD)						LGCM With Group as Covariate	
	T1	T2	T3	Within-Group Difference^a^		Cohen *d* (95% CI)^b^	T1	T2	T3	Within-Group Difference^a^		Cohen *d* (95% CI)^b^	Estimate (SE) for Slope	*P* Value
				*F*	*P* Value					*F*	*P* Value			
Cognition	53.80 (20.63)^c^	49.18 (21.15)^d^	42.38 (19.54)^c^^,^^d^	*F*_2.0,48.5_ = 9.02	<.001	−0.65 (−1.08 to −0.18)	54.46 (21.46)	54.64 (24.20)	49.20 (20.37)	*F*_2.0,48.5_ = 1.86	.17	−0.22 (−0.64 to 0.21)	−2.70 (2.11)	.20
Mobility	45.72 (26.34)^c^	35.37 (27.03)	29.73 (22.57)^c^	*F*_2.0,45.4_ = 11.42	<.001	−0.72 (−1.18 to −0.26)	41.23 (25.70)	41.82 (27.17)	38.78 (26.16)	*F*_2.0,46.4_ = 0.46	.63	−0.11 (−0.55 to 0.33)	−5.59 (2.34)	.02
Self-care	37.04 (20.98)	35.65 (23.06)	30.26 (18.67)	*F*_2.0,43.7_ = 2.01	.15	−0.43 (−0.89 to 0.07)	41.32 (23.61)	43.59 (26.80)	38.14 (27.80)	*F*_2.0,40.3_ = 1.84	.17	−0.14 (−0.57 to 0.30)	0.47 (2.44)	.85
Getting along	56.70 (25.15)	54.61 (25.94)	53.05 (22.80)	*F*_2.0,46.0_ = 0.67	.52	−0.16 (−0.64 to 0.31)	60.76 (25.90)	60.00 (27.16)	56.91 (25.75)	*F*_2.0,40.4_ = 1.77	.18	−0.16 (−0.62 to 0.31)	1.08 (2.37)	.65
Domestic responsibilities	66.36 (28.43)	54.58 (26.65)	53.75 (31.68)	*F*_2.0,43.6_ = 3.23	.049	−0.42 (−0.90 to −0.03)	66.60 (30.57)	63.81 (34.00)	57.73 (35.89)	*F*_2.0,44.6_ = 2.48	.10	−0.28 (−0.71 to 0.16)	1.03 (3.40)	.76
Participation	61.39 (19.61)^c^	55.59 (15.60)	53.70 (19.18)^c^	*F*_2.0,40.4_ = 3.79	.03	−0.52 (−0.97 to −0.06)	60.76 (21.54)	59.98 (21.59)	58.53 (20.41)	*F*_2.0,34.5_ = 0.83	.44	−0.09 (−0.56 to 0.40)	−0.59 (1.98)	.77

Abbreviations: BA, brief admission; LGCM, latent growth curve modeling; T1, baseline; T2, 6-month follow-up; T3, 12-month follow-up; WHODAS II, World Health Organization Disability Assessment Schedule II.

^a^ Outcomes analyzed through hierarchical linear modeling with time as a fixed effect.

^b^ Cohen *d* values were calculated as within-group time effect sizes from T1 to T3 using the mean difference divided by the sample T1 SD and by taking the correlation between the 2 repeated measures into account, as suggested by Morris and DeShon.^32^

^c^ Significant difference between means found from T1 to T3.

^d^ Significant difference between means found from T2 to T3.

Regarding NSSIs, there was no significant change in the number of NSSI acts for the BA group (BA group vs control group: LGCM, −0.41; *z* = 1.70; *P* = .09) (Table 3). However, a significant within-group decrease over time was found for the BA group (χ^2^ = 6.13; *P* = .047).

### Adverse Events

The stopping guidelines were not effectuated; namely, no individuals in the BA group showed escalating self-harm or suicidality, and only 1 endangered others in the ward. During the trial, 3 individuals died by suicide; 2 were randomized to the control group, and 1 was randomized to the BA group. The individual in the BA group died by suicide after randomization but before the negotiation for a BA contract; thus, none of these participants accessed the intervention.

Overall, 9 participants in the BA group experienced adverse events during BA; 1 participant had 9 adverse events, 2 had 3 adverse events, and the remaining 6 had 1 adverse event each. In total, 21 adverse events were registered; 11 were minor and lacking in suicidal intent (eg, superficial wrist cuts, punching the wall, or doubling sleep medication), 5 were related to substance use, 3 were related to an eating disorder (eg, not eating during BA or showing uncontrollable bulimic behavior), 1 communicated imminent suicidality, and 1 endangered others in the ward.

## Discussion

In this RCT, no significant differences were found between the BA and control group regarding days admitted to the hospital, compulsory admission and measures, and visits to an emergency department. Both groups showed a significant decrease in the number of days admitted to the hospital and visits to an emergency department. Only the BA group showed a significant decrease in days with compulsory admission; however, this did not yield a significant difference in the between-group analyses.

The results of the present study are in line with the findings of 2 other studies that explored the association of brief patient-controlled admissions with inpatient services,^3,4^ which strengthens the external validity. However, there were substantial differences between these studies and the present study with respect to the target population (ie, severe mental illness, mainly schizophrenia and bipolar disorder, vs self-harm and suicidality) and study design (eg, matched prospective cohort study in Thomsen et al^4^). Arguably the most important difference between the present study and the 2 previous studies is that the present study used a fixed treatment protocol for the intervention and had explicit efforts to ensure treatment fidelity, aiming at promoting replicability. However, the results yielded from the Norwegian^3^ and Danish^4^ studies and the present study were similar, indicating that BA by self-referral does not influence use of inpatient services or compulsory measures.

The BA group showed a significant improvement in several domains of functioning, which was not found in the control group. However, only the mobility domain, which involved moving and getting around outside the home, reached significance in the between-group analyses. As an overarching goal of the standardized BA was to increase autonomy, these findings align with the core aims of the intervention. As this was the only significant between-group finding, it might be an artifact. However, significant improvements were found in the BA group but not in the control group in 4 of the 6 WHODAS domains. Thus, a likelier assumption is that there was a true improvement in functioning in the BA group; the fact that it was only significant for the mobility domain might have been fortuity.

Adverse events during BA were registered and were expected because the intervention was not considered as a form of treatment but rather a protocol for managing crises. A complete termination of self-harming behaviors was not a realistic expectation, as the target population included the most severely ill individuals in the region who had participated in a wide variety of TAUs (both previous and ongoing). Nevertheless, a decline in self-reported NSSIs was found in the BA group but not in the control group, suggesting that the adverse events could not be attributed to the intervention. Due to abundant missing values, the significant decline in attempted suicide in the BA group should be interpreted with caution.

### Limitations and Strengths

This study had limitations. The sample was referred and not consecutive, which might hamper the generalizability. Another limitation was the diverse range of TAUs. However, the use of various TAUs is common in clinical situations, and taken together with the fact that 125 of the 129 referred individuals were allocated, it suggests that the study has good ecological validity. Furthermore, although measures were taken to avoid contamination between groups as much as possible, all psychiatrists at the clinics had to be informed of the study methods, which might have influenced the TAU and improved the control condition. These limitations might have attenuated the significance of results. Additionally, we could not correct for baseline group differences in anxiety. Another limitation was the predominantly female sample, although it was consistent with the sex distribution of individuals seeking help for self-harm.

The Danish and Norwegian studies,^3,4^ like the current study, explored the association of BA by self-referral with similar primary outcome measures—particularly, the use of inpatient services—supposedly based on previous studies without controls and showing large effects.^2^ Further, the target populations in all 3 studies were individuals with extensive experience of hospital admissions at the time of recruitment. Therefore, since these baseline data might be extreme, the results might be better explained by a regression toward the mean than by a true effect of the intervention.^34^ There is also a possibility of contamination between the intervention and control conditions. Further, as noted above, BA is not a treatment but rather a way of organizing hospital admissions in times of crisis; this suggests that a dramatic improvement in symptomatology and a consequent reduction in the need for hospital admissions cannot be expected, solely due to having access to the intervention. It might also be that 12 months is too short a period for follow-up to yield significant between-group differences.

The choice of primary outcome measure (number of days with inpatient services and compulsory measures) might have been lacking dimensions relevant for mental health care, such as the experiences of both users and health care professionals. Qualitative aspects found in previous studies,^2,4^ such as the perceptions of individuals using and providing BA, have not been explored and would preferably be addressed with a qualitative design.

## Conclusions

To our knowledge, this is the first RCT to evaluate the effect of patient-controlled BA for individuals who recurrently self-harm and are at risk of suicide on inpatient service use, daily life functioning, NSSI, and attempted suicide. In this study, access to BA by self-referral did not influence the use of inpatient services or compulsory measures. Other possible beneficial effects should be further explored.

## Notes

Supplement 1. **Trial Protocol**

Supplement 2. **eTable 1. **Strategies Applied in Brief Admission Skåne Randomized Controlled Trial (BASRCT) for Enhancing Treatment Fidelity **eTable 2. **Growth Curve Estimates for Total Days Admitted to Hospital **eTable 3.** ZIP Growth Curve Modeling Estimates for the Count Variables **eTable 4.** Growth Curve Estimates for the WHODAS Domains **eFigure 1.** The Number of Days With General Admission to Hospital, With Compulsory Admission to Hospital, and With BA for the BA Intervention Group (A) and for the Control Group (B) Over 3 Time Points **eFigure 2.** The Mean Scores for the WHODAS Domains at 3 Time Points for the BA Intervention and Control Groups

Supplement 3. **Data Sharing Statement**
