# Error in Table

**DOI:** 10.1001/jamanetworkopen.2019.13724

**Published:** 2019-10-04

The Original Investigation titled “Effect of Brief Admission to Hospital by Self-referral for Individuals Who Self-harm and Are at Risk of Suicide: A Randomized Clinical Trial,”^1^ published June 7, 2019, included an error in Table 3. The second row was labeled “Days with general admission,” and it should have been labeled “Days admitted to hospital, excluding BA.” This article has been corrected.^1^
