# Supplementary material for: Effect of Brief Admission to Hospital by Self-referral for Individuals Who Self-harm and Are at Risk of Suicide: A Randomized Clinical Trial
Source: JAMA Netw Open. 2019 Jun 7;2(6):e195463. doi: 10.1001/jamanetworkopen.2019.5463 (PMC6563573; doi:10.1001/jamanetworkopen.2019.5463)
Supplement: Supplement 1. — Trial Protocol [file jamanetwopen-2-e195463-s001.pdf]

eAppendix1. Trial protocol

BRIEF ADMISSION SKÅNE: REPLACING GENERAL ADMISSION FOR INDIVIDUALS WITH SELF-HARM AND ACUTE RISK OF SUICIDE.

The Brief Admission Skåne Randomized Controlled Trial (BASRCT)

Aims:

The overall aim of this study is to determine whether Brief Admission Skåne (BA) can replace General Admission (GA) to hospital, for individuals that self-harm at risk for suicide.

Primary Research Question:

1. Can BA replace GA for individuals with self-harm at acute risk for suicide?

Secondary Research Questions:

2. Can BA increase the individual's level of functioning in activities of daily life?
3. Can BA increase the individual’s ability to cope effectively with life stress?
4. Can BA reduce the individual's global psychiatric symptoms?
5. Can BA reduce the frequency of all self-harming behaviours including suicide attempts?
6. Can BA reduce the severity of self-harming behaviours?
7. Can BA serve as feasible management model in the care of individuals with self-harm, who may also be at risk for suicide?
8. Can the *Five Self-Harm Behaviour Groupings Measure* reliably and validly measure behaviors ranging from indirect to direct self-harm and attempted suicide, with varying degree of frequency and severity?

Area overview:

A recent study examining the prevalence of self-harm in psychiatric settings in Sweden, found that almost half of the individuals currently receiving mental-health services had self-harmed during the past six months (Odelius & Ramklint, 2014). Of those who had engaged in self-harming behaviour, more than 90% have had suicidal thoughts during their life-time and more than every other had at least once during their lives attempted suicide. For a small group of individuals, acts of self-harm are frequent and risk for suicide is recurrent, prolonged and high (Lieb, Zanarini, Schmahl, Linehan & Bohus, 2004). Many of these individuals are diagnosed with Borderline Personality Disorder (BPD).

Over the last 20 years several psychotherapeutic interventions have evolved for the treatment of individuals with self-harm as well as BPD (Lundh, 2014; Stoffers, Völlm, Rucker, Timmer, Huband & Lieb, 2012). However, when these individuals seek acute admission to hospital due to a crisis and associated increased suicidal ideation, recommendations for clinical care are still conflicting. For individuals with any other kind of diagnosis and severe suicidal ideation, the routine is to offer acute admission to an inpatient unit. However for individuals with recurrent suicidal ideation and self-harm, the risk for iatrogenic effects are considerable, and long hospital admissions without a clear treatment structure may predict decompensation in functioning (Lundh, 2013; Lundh 2014). This has resulted in a clinical practice of avoiding admission for individuals diagnosed with BPD.

These two obviously conflicting recommendations can be hazardous for individuals seeking help due to imminent suicidal crises and provide a regular and ongoing source of stress for staff at psychiatric emergency wards. They create conflict among all specialized mental health service providers who share the clinical responsibility to preserve the life of acutely suicidal individuals at the very moment that smooth transitions from outpatient to inpatient care are vital. The contradictory recommendations are a regular burden requiring strategic management at junctures that would be better suited to the provision of clinical care.

Brief Admission (BA, Brukarstyrd inlägging) is a model in which the individual seeking psychiatric care can decide themselves when they need hospital admission to prevent decompensation of mental health functioning, including suicidality, for a short period (days) at a maximum frequency (admissions per month). The model has been used in the Netherlands for more than 30 years but has not yet been scientifically evaluated in controlled trials.

A recently-published review article examined the key elements that are fundamental for effective short-term admissions of individuals with prolonged suicidality, self-harm or BPD (Helleman, Goossens, Kaaseenbrood, & van Achterberg, 2013). The number of publications in indexed journals was found to be limited with different study designs, however five key elements of BA emerged:

1. In advance, a discussion of the goals with the BA. Possible targets with the BA might be to prevent long-term hospitalization, reducing the number of acts of self-harm/ suicide attempts, to prevent power struggles between individuals seeking care and care providers, facilitating the return to ambulatory care, and to offer an admission which does not reduce the individual's autonomy by being unstructured and of unpredictable or too-lengthy duration.

2. To provide a clear admission procedure. Prior to the BA a personal, written agreement in the form of a contract concerning the time frame and goal of the admissions. In the reviewed studies possible admissions varied between 3 and 14 days and "refractory periods" between 14 and 30 days.

3. The individual seeking BA should have clear instructions regarding how to predictably access an admission at the time it is needed.

4. Specification of which interventions are accessible and which interventions are not accessible during the BA. This should be defined in prior to the BA. The type of interventions varied between studies from conversations with nurse (5 studies) to varying degrees of assessment and treatment. This specification is also necessary to distinguish between the BA and a regular clinical admission.

5. Five out of ten studies had predefined conditions for premature, involuntary discharge. These conditions were in all studies, individually-tailored to address the circumstance of the individual. Such conditions, however, are controversial, since several of those tested (expression of suicidality, intoxication, self-harm) are signs that the individual in crisis needs to be taught skills that would reduce reliance on these behaviours which they themselves often find undesirable (Linehan, 1993).

A Dutch study examined individuals with BPD and a history of long hospital admissions (Koekkoek, van der Snoek, Oosterwijk &van Meijel, 2010). Only eleven (N=11) participants were included. The participants were offered voluntary, planned admissions to hospital over a period of six months. The amount of days they were offered was estimated from how much they had been admitted to hospital during the previous six months. The quality of the therapeutic relationship was rated by asking the professional to rate the degree of agreement between participant and professional on content and form of the treatment by using a seven-point Likert scale with 1 indicating a complete lack of agreement and 7 indicating perfect agreement. Over the course of the intervention, the ratings increased substantially and significantly, and services use decreased substantially, yet not significantly (possibly due to the small sample size). Participants expressed feeling very content with the intervention.

Koekkoek (2010) stresses the importance of ensuring that the conditions of the contract and

aims of the BA are thoroughly discussed with the individual and her/his ambulatory clinician. This has two purposes - first, the individual needing care feels that the intervention fully is her/his choice, and thereby increasing responsibility and autonomy, and secondly the collaborative discussions in which the individual's perspective influences decisions about their care will build the therapeutic alliance (Koekkoek, 2010).

A Norwegian study included 24 individuals with mixed diagnoses and extensive use of hospital admission (Støvind, Hanneborg, Ruud, 2012). Eight of the individuals had schizophrenia, and the remaining participants had affective disorder (n=7), anxiety disorder (n=4), personality disorder (n=3), substance abuse (n=1) or lacked a diagnosis (n=1). The participants could themselves decide when they wanted to be admitted to hospital, and stay for durations of up to five days. After an admission period, they had to be treated in an ambulatory setting for at least 14 days before they again had the opportunity to choose another five-day admission. The total number of participants was small and the study made no estimates of significance, but the number of involuntary admissions was halved, and participants reported feeling more satisfied with their care when brief admissions were included. For participants with schizophrenia, the number days of hospital admission did not change with the intervention possibly due to the course of acute psychosis. However, for the remaining sixteen participants, hospital admission decreased from 37% of the days during the six months preceding the intervention, to 13% of the days during the six months of intervention. The frequency of admissions increased, but each admission lasted on average only two to three days.

Description of the project

Sites:

Psykiatri Skåne provides inhabitants in Region Skåne with psychiatric healthcare. Region Skåne has about 1,3 million inhabitants and is served by four geographically organized psychiatric divisions (Helsingborg, Kristianstad, Lund and Malmö) and two that are organized by content (Child and Adolescent Psychiatry and Forensic Psychiatry). The geographically based divisions are served by four inpatient settings (Helsingborg, Kristianstad, Lund and Malmö) and several ambulatory units. About 3000 people are currently employed at Psykiatri Skåne. Researchers conducting the study are based in Lund (Sofie Westling, Sophie Liljedahl, Daiva Daukantaité and Åsa Westrin) where the administrative center of Region Skåne also is located, and in Groeningen, the Netherlands (Marjolein Helleman). The pilot phase of the study will take place in Lund and Malmö.

Definitions

Brief Admission (BA – Brukarstyrd inläggning) is in this project defined as the specific intervention, standardized by the Brief Admission Skåne Fidelity Measure (BASFM, Bilaga 2b; Liljedahl, Helleman, Daukantaite & Westling, 2017). *Brief Admission Skåne* (BAS - *Brukarstyrd inläggning Skåne*) is the randomized controlled trial evaluating the intervention.

General Admission (GA – Läkarstyrd inläggning) is defined as all other admissions, voluntary as well as coercive, to the emergency ward (psychiatric or somatic) due to psychiatric needs or following an act of self-harm or a suicide attempt, including possible following days with hospital admission.

Patient selection criteria

Inclusion criteria:

- Current episodes of self-harm and/or recurrent suicidality.

- Fulfilling at least three criteria for Borderline Personality Disorder.
- Admitted to hospital care for at least 7 days or presenting to the psychiatric emergency department at least 3 times during the last six months.
- Age 18-60 years.

Exclusion criteria:

- No current ambulatory clinician
- No current place to live (homeless).
- Medical disorder from other organs that significantly contributes to symptoms (e.g. if self-harm only occurs during episodes of hypogylcemia in a diabetic patient).

Testing for autism, attention deficit or learning disabilities exceed the scope of this study. These diagnoses are not considered to be exclusion criteria, neither are they related in a more direct way to any of the research objectives. Thus, since the proposed assessments already are considerably time-consuming (see justification for measures, below), testing for these diagnoses is excluded.

Methods of evaluation (Figure 1, Bilaga 2c and Bilaga 5a-j)

Data collected from hospital records

From local hospital records, data is collected concerning:

- Number of days with general admission to hospital
- Visits at the psychiatric emergency department,
- Whether the admission was voluntary or coercive
- Coercive acts as defined by LPT; 1991:1128: §19, 20, 21, 22, 23 and 24.

Hospital data are collected monthly retrospectively from twelve months before the intervention start of the pilot and until endpoint after 12 months of the active study period. This generates quantitative data registered in a form (Bilaga 5a).

Justification for Measures

The self-report and clinician-administered assessments in this section were included after careful and repeated consultation within the research group, balancing sensitivity to the needs of the individuals with the aim to answer the research questions at the core of this study. The shortest and most concise versions of the measures were selected, and the frequency of assessment intervals (see the Design section below) was specifically chosen to reduce the burden to the individual when completing the measures of the study.

Included in the protocol are three different self-harm measures, two of which are self-report, and one that is clinician-administered. Although the same behaviour (self-harm) is ultimately being queried, both self-report self-harm measures evaluate different, non-overlapping aspects of the behaviour. The clinician-administered self-harm measure is being validated in this study for use in clinical samples (Liljedahl & Westling, 2014). It is based upon a broad definition of self-harm that involves querying self-harm that is direct, indirect, lower-to-higher severity and lower-to-higher lethality, including suicide attempts. If this measure does prove to be reliable and valid, then future researchers and clinicians can use it rather than self-report measures based on narrow self-harm definitions that do not reflect the nature of severe and repetitive self-harm that can and does escalate into suicidal behaviour for some individuals.

Two additional steps included in this protocol to respond to the sensitivity of individuals in distress, are:

1. To ensure that individuals are not left on their own while completing self-report measures. They will be in the presence of an experienced research nurse who can support and encourage them to take breaks or discuss any items with which they might struggle.

2. To pilot the evaluation measures and the new self-harm measure (5S-HM) with individuals that have lived experience of self-harm, as well as the significant others in their lives through the Swedish voluntary organization SHEDO (self-harm and eating disorders organization: www.shedo.org). Candidates from SHEDO have already agreed to participate in the piloting of these new measures. Their feedback will be integrated to the phrasing of the new measures as well as the manner in which they are administered.

Self-report measures/ evaluations

The Brief COPE (Carver, 1997) is a self-rating scale that describes coping strategies to handle stressful situations within the areas of self-distraction, active coping, denial, alcohol/drugs, use of emotional support, use of instrumental support, behavioural disengagement, venting, positive reframing, planning, humour, acceptance, religion and self-blame. Each area is covered by two items (totally 28 items) to which the individual responds on a Likert scale with four possible answers covering from "I haven't been doing this at all" to "I've been doing this a lot" (Carver, 1997, attached in Bilaga 5c). The Swedish version of the Brief COPE (Muhonen & Torkelson, 2005) will be used in the proposed study (Bilaga 5c).

The Difficulties in Emotion Regulation Scale (DERS; Gratz & Roemer, 2004) is a questionnaire developed to measure difficulties in emotion regulation. It consists of 36 items rating six different dimensions of emotion regulation. Response items are presented on a Likert scale with five possible answers ranging from "almost never" to "almost always." Higher scores indicate more difficulties. The Swedish version of the DERS (Friberg, 2006) will be used in the proposed study (attached in Bilaga 5d)

The Inventory of Statements About Self-injury (ISAS; Klonsky & Glenn, 2009; Glenn& Klonsky, 2011) is a self-rating measure on self-harming beahviour (Swedish translation: Lindholm, Bjärehed & Lundh, 2011). Is contains questions concerning the frequency of 12 different forms of self-harm as well as 39 statements about the functions of self-harm, using a three-point Likert scale, ranging from "0-not relevant," "1-somewhat relevant," to "2-very relevant". Five additional questions assess descriptive and contextual factors, including age of onset, the experience of pain during, whether the person is alone or around others when self-harming, time between the urge to self-harm and the act, and whether the person wants to stop self-harming.

The World Health Organization Disability Assessment Schedule II (WHODAS 2.0; 2014) is a self-rating questionnaire developed by the World Health Organization (WHO), in which the participant responds to 36 questions investigating level of functioning and disability in the domains of cognition, mobility, self-care, getting along with other people, life activities and participation in community activities. The questionnaire is undergoing an authorized translation from English to Swedish conducted by Socialstyrelsen that will be finished during autumn 2014. Cecilia Svanborg and Kristina Brand-Persson (Cecilia.Svanborg@ki.se resp. Kris-

tina.Brand-Persson@socialstyrelsen.se) who are responsible for the translation, certify that there will be no significant deviations in content during the translation from English to Swedish. (Attached is the English version, Bilaga 5b)

Individual's Experience Scale (IES; Liljedahl, Helleman, Daukantaite & Westling, 2017; attached in Bilaga 5h) is an evaluation form derived from the Brief Admission Skåne Fidelity Measure (BASFM; Liljedahl, Helleman, Daukantaite & Westling, 2017). The IES is aimed for the individual receiving BA, and investigates 6 different domains of BA, covered by 31 statements, to which the individual responds using a four-point Likert scale, ranging from 0 – do not agree at all to 3 - agree completely. The CES is aimed for the clinician, delivering the BA, and investigates the same six domains of BA, in the same manner but targeting the clinicians' experience.

Clinician's Experience Scale (CES; Liljedahl, Helleman, Daukantaite & Westling, 2017; attached in Bilaga 5i) is the second evaluation form derived from BASFM; (Liljedahl, Helleman, Daukantaite & Westling, 2017). The CES is aimed for the clinician, administering BA, and investigates 6 different domains of BA, covered by 35 statements, to which the individual responds using a four-point Likert scale, ranging from 0 – do not agree at all to 3 - agree completely.

The *Alcohol Use Disorder Identification Test* (AUDIT; Saunders, Aasland, Babor, Delafuente, Grant, 1993; Babor, Higgins-Biddle, Saunders & Monteiro, 2001) is a self-report questionnaire, developed by the WHO, covering alcohol use patterns and related problems (total score range 0–40, higher scores indicating a greater degree of risk). It is considered the gold standard test for screening for alcohol use disorders, is widely used internationally and has been translated to Swedish (Bergman & Källmén, 2002 .

The *Drug Use Disorders Identification Test* (DUDIT), with proven reliability and validity (Berman, Bergman, Palmstierna & Schlyter, 2005), measures use of illicit drugs and drug-related problems (total score range 0–44, higher scores indicating a greater degree of risk, harm or intensity).

The *Client Satisfaction Questionnaire* (CSQ; Nguyen, Attkisson, & Stegner, 1983) is one of the more widely used measures investigating client satisfaction with human services (Socialstyrelsen, 2013). We use the 8 items version with score range from 8 to 32, higher values indicating a higher degree of satisfaction.

Five Self-Harm Behaviour Groupings Measure (5S-HM; Liljedahl & Westling, 2014) is an instrument developed to assess and grade a wide range of self-harming behaviour, including direct and indirect self-ham, ranging from lower to higher severity and lethality, both with or without suicidal intent. Scoring criteria are included, with higher scores indicating greater severity and frequency of self-harm. Clinical cut-offs will be established over the course of the pilot phase and the psychometric validity of the measure will be tested based on data collected in this study. (Attached in Bilaga 5f).

Fem frågor (Holmqvist & Nylander, 2013a; Bilaga 5j) is a screening tool to detect developmental cognitive disabilities, such as ADHD, learning disability or autism, in individuals seeking help for psychiatric symptoms (Holmqvist & Nylander 2013b; Bilaga 5k). Answer

yes on any of the first four questions or no on question number 5 may be a sign of the presence of a developmental cognitive disability. This measure is not validated but brief and is aimed to complement the other diagnostic measures since they do not screen for developmental disabilities.

Additional questions are asked on demographics, current psychological and pharmacological treatment, as well as other interventions and assistance from the municipality.

Clinician-administered interviews

The Mini-International Neuropsychiatric Interview (M.I.N.I. 7.0.0; for Diagnostic and Statistical Manual of Mental Disorders, Fifth Edition (DSM-5); Copyright 1992---2014 Sheehan DV; Swedish version personal communication by Allgulander C.). M.I.N.I. 7.0.0 is a short, structured diagnostic interview assessing psychiatric disorders of axis I in DSM-IV and ICD-10. It is widely used and has been validated against Structured Clinical Interview for DSM diagnoses (Sheehan, Lecrubier, Harnett-Sheehan, Janavs, Weiller, Bonara, Keskiner, Schinka, Knapp, Sheehan & Dunbar, 1997) and the Composite International Diagnostic Interview for ICD-10 (Lecrubier, Sheehan, Weiller, Amorim, Bonora, Sheehan, Janavs & Dunbar, 1997). The Swedish version will be used (translation by Allgulander, C Wærn, M., Humble, M., Andersch, S., Ågren, H.)

Structured Clinical Interview for DSM IV Axis II disorders (SCID II; First, Gibbon, Spitzer, Williams & Benjamin, 1998)**.** SCID II is the most widely used diagnostic measure to determine personality disorders (axis II in DSM IV). It consists of 119 initially self-administered questions to which the respondent can answer yes or no. After the participant has completed the clinician uses the questionnaire as a base for a structured interview revealing if reported symptoms are of clinical significance or not.

Clinical Global Impression Severity (CGI-S; Guy, 1976). In CGI-S the clinician rates the severity of the patient's illness according to a seven-point scale where every number is predefined and ranging from 1 signifying normal to 7 signifying extremely ill (relative to the clinician's past experience with patients who have the same diagnosis). The Swedish version of the scale is not validated (translation: Adler, M., Agestam, M., Bergman, L., Båve, U., Nordlund, S., Norring, C., Rosenqvist G., 2010) but commonly used when assessing symptom severity and treatment response in individuals with mental disorders. (attached in Bilaga 5e).

Study design

The design for this project will be a Randomized Control Trial (RCT), combined with Time-series (TS; Borckardt et al, 2008). Participants will be randomized at an individual level to either BA + Treatment as Usual (TAU) or TAU. Block randomization, using tables with random numbers with blocks of four, will be used in order to minimize the confounding effect of changes in general care over time. Randomization will be stratified according to site (i.e. Lund, Malmö, Kristianstad, Helsingborg). Random number tables will be generated in SPSS. The data will be handled with Intention to treat (ITT) analysis, so that once participants are randomized, their data will be included in all analyses regardless of whether they drop-out of the study prior to its termination.

Every participant receives a consecutive research number indicating to which site they belong (Lund – L01-…, Malmö – M01 - …; Kristianstad – K01-…, Helsingborg – H01-…). The researcher who is methodologically responsible prepares the randomization lists in four series

(one per site). The randomization numbers are named according to strata (Lund – LR01-…, Malmö – MR01 - …; Kristianstad – KR01-…, Helsingborg – HR01-…). From the lists, a research nurse prepares four series of consecutively numbered, sealed, opaque envelopes, each containing information on which group the participant is randomized to. After inclusion in the study, the research participant is given the envelope to open. After reading, PI handles a letter containing information on which upcoming procedures for the group to which the participant has been randomized. The randomization enveloped is handled back to PI and stored in a locker, together with the master key.

In the master key, PI register the name of the participant in combination with the research code and the randomization code. Data from forms are collected online, encoded by the research number, and stored on Lund University server. These assessments will be blinded to researchers analyzing the data. Videos and audio-recordings will be stored on USB in a locker separate from the master key. Recordings will only be reviewed on computers not connected to Internet.

All participants randomized to the intervention during the pilot phase as well as the 10% of the individuals with the highest number of days admitted to hospital 12 months before baseline, will be selected for Time-Series Design (TS). The TS will follow an A-B replication case-series design where the number of days of GA to hospital will be monitored monthly, retrospectively with data from the local hospital records, from one year ahead of assigning to the study (A) and during the time the participant is allocated to either BA+TAU or TAU (one year; B).

Testing Schedule

For a visual description of the testing schedule, please see Figure 1, Bilaga 2c.

1. Individuals with symptoms suggesting that they may fulfill inclusion criteria, are asked by any clinician at the current department, if they want to participate in the study. If the individual is interested in participating, the clinician passes contact information to the PI.
2. PI checks if the inclusion criteria seem to be fulfilled and no exclusion criteria.
3. PI provides written and verbal information about the study, including time for questions and asks the individual to sign the consent form.
4. PI registers the participant in the screening log and provides a consecutive research number.
5. PI performs assessments with:
    a. M.I.N.I. 7.0.0 (Sheehan, Lecrubier, Harnett-Sheehan, Janavs, Weiller, Bonara, Keskiner, Schinka, Knapp, Sheehan & Dunbar, 1997; Lecrubier, Sheehan, Weiller, Amorim, Bonora, Sheehan, Janavs & Dunbar, 1997)
    b. SCID II (First, Gibbon, Spitzer, Williams & Benjamin, 1998)
    c. Fem frågor (Holmqvist & Nylander, 2013a),
    d. AUDIT (Saunders, Aasland, Babor, Delafuente, Grant, 1993; Babor, Higgins-Biddle, Saunders & Monteiro, 2001)
    e. DUDIT (Berman, Bergman, Palmstierna & Schlyter, 2005).
6. PI provides a consecutively numbered randomization envelope which the participant opens and signs. This is registered in the randomization log. According to which

group the participant is enrolled in, a sheet providing information on the study is given and explained.

7. Data collection at baseline by PI:
   a. For the individuals randomized to the control group, data from hospital records for the previous 12 months, and CGI-S (Guy, 1976), are recorded with baseline date on the day for the randomization.
   b. For individuals randomized to the intervention group data from hospital records and CGI-S (Guy, 1976), are recorded with baseline date on the day for the contract (i.e. day when BA is accessible).
8. Data collection at baseline by a Research Assistant (RA):
   a. After randomization PI contacts a local RA who schedules an appointment with all participants, and administers a link to the self-administered forms:
      i. 5S-HM (Liljedahl & Westling, 2014),
      ii. WHODAS 2.0 (WHODAS 2.0; 2014),
      iii. Brief COPE (Carver, 1997; Muhonen & Torkelson, 2005), DERS (Gratz & Roemer, 2004; Friberg, 2006),
      iv. ISAS (Klonsky & Glenn, 2009; Glenn& Klonsky, 2011; Lindholm, Bjärehed & Lundh, 2011) .
   b. RA is available for the individual and provides help if necessary, when completing the forms online.
   c. For individuals randomized to the intervention group RA further contacts their primary clinician as well as ward staff at the ward providing BA, to schedule an appointment for negotiation resulting in a BA contract. At the end of the negotiation IES and CES (negotiation part) are completed online.
9. Datacollection as 6 and 12 months (+/-2 weeks) is repeated as baseline, with the change that the contract negotiation is replaced by contract evaluation for the intervention group and data from hospital records is collected from the previous 6 months.

Pilot phase

The first three months of the study (Sept, 2015 – Jan, 2016) will form a pilot phase with the goal of optimizing the intervention, evaluate the inclusion and exclusion criteria and preliminary testing to determine whether the quality and quantity of assessments are adequate and feasible. At the termination of the pilot phase evaluation with IES and CES (Liljedahl, Helleman, Daukantaite & Westling, 2017) will be performed.

Data collection will be suspended from January to March, 2016. During this phase all audiotaped sessions will be transcribed, translated and evaluated by the authors of the BASFM (Bilaga 2b; Liljedahl, Helleman, Daukantaite & Westling, 2017), Sophie Liljedahl and Marjolein Helleman. Feedback from the evaluation measures (the IES and CES, Liljedahl, Helleman, Daukantaite & Westling, 2017) will be extracted and reviewed by the senior researchers in this study to determine whether the content or procedures are functioning as anticipated, and to determine whether there are any areas in need of improvement. Any substantial changes to any aspect of the study or its measures will be sent to the Regional Ethics board (EPN Lund) for review. Data collection for the active phase of the study will start between March 2016 (baseline) and terminate 36 months after.

Intervention and Treatment as Ususal (TAU):

Participants randomized to TAU will receive no intervention from the study protocol, except the baseline assessments and repeated assessments administered on the same schedule as described above for the treatment group. They will not be given the evaluation measures that are specific to the BA intervention (the IES and the CES; Liljedahl, Helleman, Daukantaite & Westling, 2017)

For a detailed description of the BA please see the *Brief Admission: Manual for training and implementation developed from the Brief Admission Skåne Randomized Controlled Trial* (Liljedahl, Helleman, Daukantaite & Westling, 2017). Participants randomized to BA + TAU will have an initial, scheduled meeting with their ambulatory clinician and the local RA who also functions as the nurse clinician as described in the BASFM (Liljedahl, Helleman, Daukantaite & Westling, 2017). During this meeting the participant receives further information on BA. This meeting comprises a negotiation process as standardized in the BASP with the goal of integrating the intervention in the individual's treatment plan. The meeting results in the BA contract, signed by the participant, their ambulatory clinician and the local RA (attached in Bilaga 4c). The ambulatory clinician stays responsible for the treatment and the RA becomes the contact person to bridge the gap between ambulatory care and the BA.

A defining feature of the BA is that the participant decides for themselves regarding whether and when to initiate a brief hospital admission, at most three times per month, and for a maximum of three consecutive days. BAs can be initiated between 8AM and 8PM every day during the week. The procedure for the BA is defined in the BASP. If the needs of the client exceed the level of service offered during BA, GA should be considered.

The intervention will last for 12 months and the participant will evaluate the intervention at the end of each BA and the contract after 6 and 12 months according to the procedure as described in BASFM (Bilaga 2b; Liljedahl, Helleman, Daukantaite & Westling, 2017). Participants randomized to the BA condition have access to the same security procedures as they do when receiving Treatment as Usual (TAU).

No risks to the well-being of the participant are expected, exceeding those found with TAU. Normal security routines are therefore sufficient. Data collection will start in September 2015 for the pilot phase of the study, and evaluated at the end of January 2015.

How is the choice of methods related to the Research Objectives?

1. Can BA replace GA for individuals with self-harm and acute risk for suicide?
   – Outcome is data from medical records.

2. Can BA increase the individual's level of functioning in activities of daily life?
   – Outcome is data from WHODAS 2.0. (World Health Organization, 2014).

3. Can BA increase the individual's ability to cope effectively with life stress?
   – Outcome is data from Brief COPE (Carver, 1997) Swedish version of the Brief COPE (Muhonen & Torkelson, 2005) and DERS (Gratz & Roemer, 2004); Swedish version of the DERS (Friberg, 2006).

4. Can BA reduce the individual's global psychiatric symptoms?
   – Outcome is data from estimation according to CGI-S (Guy, 1976).

5. 5. Can BA reduce the frequency of all self-harming behaviours including suicide attempts?

- Outcome is data from questionnaire 5S-HM (Liljedahl & Westling, 2014) and ISAS (Klonsky & Glenn, 2009; Glenn& Klonsky, 2011; Lindholm, Bjärehed & Lundh, 2011).

6. Can BA affect the severity of self-harming behaviours including suicide attempts?
   - Outcome is data from questionnaire 5S-HM (Liljedahl & Westling, 2014).

7. Can BA serve as a feasible management option in the care of individuals with self-harm who may also be at risk for suicide?
   - Outcome is data from IES and the CES (Liljedahl, Helleman, Daukantaite & Westling, 2017) completed by the participants and clinicians administering BA after pilot testing, 6 and 12 months

8. Can the *Five Self-Harm Behaviour Groupings Measure* (5S-HM: Liljedahl & Westling, 2014) reliably and validly measure behaviors ranging from indirect to direct self-harm and attempted suicide, with varying degree of frequency and severity?
   - Outcome is data from questionnaire 5S-HM (Liljedahl & Westling, 2014), ISAS (Klonsky & Glenn, 2009; Glenn& Klonsky, 2011); Swedish version of the ISAS (Lindholm, Bjärehed & Lundh, 2011) and DERS (Gratz & Roemer, 2004); Swedish version of the DERS (Friberg, 2006).

Required sample size and a priori power analyses

G*Power, 3. 1. 7 (Faul, Erdfelder, Lang, & Buchner, 2007) was used to calculate a priori power for analyzing main effects and interaction for an A X B mixed design where A is a between-subject factor with two levels (experimental and control groups) and B is a within-subjects factor with three levels (three repeated assessments). The main statistical analyses will be either mixed (within-between) Analysis of Variance (ANOVA) or Analysis of Covariance (ANCOVA), controlling for baseline. Simpler univariate analyses will be calculated, but the power analyses summarized here are for the ANOVA model.

Assuming that three effects (i.e., between levels of the factor A, within levels of the factor B and within-between interaction A X B), are of medium size (f = 0.25; see Cohen, 1988), a significance level is of $\alpha = .05$, and the power values of the F tests are .85, a total of N = 98 per treatment site must be recruited (n=196 including both Stockholm and Lund, whose data will be grouped and analyzed separately).

Attrition in this population based on previous studies has been estimated to be approximately 25% (Stoffers, Völlm, Rucker, Timmer, Huband & Lieb, 2012; Nadort, Arntz, Smit, Giesen-Bloo, Eikelenboom, Spirnhoven, van Asselt, Wensing, & van Dyck.,2009). In order to attain the required sample size for these power estimates, including expected attrition, a total of of N= 124 participants is required, with n=62 participants in each group (treatment and control).

Significance:

Although individuals diagnosed with Borderline Personality Disorder (BPD), with recurrent risk for self-harm and suicide, frequently seek help in psychiatric care, there is no consensus regarding how to they are best treated when in crisis with high risk for suicide. As is the nature of individuals diagnosed with BPD or those with pervasive emotion dysregulation, suicidality has been described as “chronic” (Linehan, 1993). Accordingly, an evidence-based model of managing suicidal crises will be a significant contribution to the care of these individuals and their care-providing network.

The variation in the care offered to these individuals is, at the moment, vast. These individuals are critically ill with mortality from suicide of approximately 10%, which is 50 times higher than in non-clinical populations (Lieb, Zanarini, Schmahl, Linehan & Bohus, 2004). Brief Admission(BA) has the potential to serve as a new strategy, offering brief and structured hospital admission with low risk of iatrogenic reinforcement of suicidal behaviour. A protocol for managing crises may reduce the stress that professionals responsible for therapeutic outcomes often experience, which has often unfortunately led to stigma of these individuals within the health and mental health care system (NICE, 2004). Reduced stress amongst attending mental health professionals may in turn result in better care, as well as a larger number of clinicians becoming willing to work with individuals with recurrent and prolonged risk for suicide and self-harm or a BPD diagnosis.

Preliminary results

None.

Previous experience:

Marjolein Helleman is a nurse and post-doctoral researcher from the Netherlands with extensive experience in working with BA. In the Netherlands BA is a well-established treatment intervention, with a history of 30 years. Among the other researchers are senior psychiatrists (Sofie Westling and Åsa Westrin) who both have experience in treating individuals with BPD at risk for self-harm and suicide. Sophie Liljedahl has a doctorate in clinical psychology based on a scientist-practitioner model, and has extensive clinical and research experience in the field. The few existing publications have not identified any risks or complications related to the intervention.

Relevant security measures

Participants will have access to the same care and security measures as before the intervention. No significant risks are expected or associated with the intervention thus no additional security measures are needed.

Ethical considerations:

Individuals with recurrent and prolonged self-harm behaviour represent a stigmatized group in health care (NICE, 2004). Lengthy and unstructured hospital admissions, which often occur in Sweden and many other countries outside of the Netherlands, have been observed to aggravate self-harming behaviour problems. Because individuals experiencing high emotional distress are largely ignored unless self-harming or suicidal, self-harming and suicidal behaviours become unintentionally reinforced when they are attended to by ward staff. Other individuals tend to observe this relationship from each other when they are in a closely-shared environment, such as an inpatient ward, and subsequently increase the frequency of their self-harming and suicidal behaviours. Ignoring these behaviours entirely has been described to be damaging and inhumane (Åkerman & Eriksson, 2011). For this reason, lengthy and unstructured hospitalizations are understood to be iatrogenic (Linehan, 1993). If BA proves to be a form of care that reinforces autonomy and responsibility for this group while avoiding the pitfalls of escalating self-harming and suicidal behaviour while in care, it could form a new model of hospital admission for this group of individuals, increasing coping skills, and providing the increased structure and care that these individuals periodically need (Helleman, Goossens, Kaasenbrood & van Achterberg, 2013).

Participation in this project is not expected to cause any physical or mental injury, pain or discomfort. During the intervention, the individual has access to their full regular treatment except when admitted to the BA. Once randomized to the BA condition, participants and have full control over whether they want to use the BA intervention. A fear that might arise in some healthcare providers is that BA could be misused. However, previous experiences from the Netherlands and Norway give no indication of this but rather the opposite (Koekkoek, van der Snoek, Oosterwijk &van Meijel, 2010; Støvind, Hanneborg, Ruud, 2012). Taken together, risks for the participants are no greater than treatment as usual, and the potential benefits are significant.

References

Adler, M., Agestam, M., Bergman, L., Båve, U., Nordlund, S., Norring, C., Rosenqvist G. (2010). Retrieved at: http://www1.psykiatristod.se/Global/Bilagor/CGI-S.pdf

American Psychiatric Association (2013). *Diagnostic and statistical manual of mental disorders* (5th ed.). Washington, DC: Author.

Babor, T. F., Higgins-Biddle, J. C., Saunders, J. B. and Monteiro, M. G. (2001). *AUDIT The Alcohol Use Disorders Identification Test. Guidelines for Use in Primary Care* (2nd ed.). Geneva: World Health Organization

Bergman, H. & Källmén, H. (2002). Alcohol use among Swedes and a psychometric evaluation of the Alcohol Use Disorders Identification Test. *Alcohol & Alcoholism, 37*, 245-251.

Berman, A. H., Bergman, H., Palmstierna, T. and Schlyter, F. (2005) Evaluation of the Drug Use Disorders Identification Test (DUDIT) in criminal justice and detoxification settings and in a Swedish population sample. *European Addiction Research 11,* 22–31

Borckardt, J. J., Nash, M. R., Murphy, M. D., Moore, M., Shaw, D., O'Neil, P. (2008). Clini cal practice as natural laboratory for psychotherapy research: a guide to case-based time-series analysis. *The American Psychologist, 63,* 77-95.

Cohen, J. (1988). *Statistical power analysis for the behavioral sciences* (2nd ed.). Hillsdale, NJ: Lawrence Earlbaum Associates.

Carver, C. S. (1997). You want to measure coping but your protocol's too long: Consider the brief COPE. *International Journal of Behavioral Medicine, 4,* 92-100.

Glenn, C. R. & Klonsky, E. D. (2011). One-year test-retest reliability of the Inventory of Statements about Self-Injury (ISAS). *Assessment, 18,* 375-378.

Gratz, K. L. & Roemer, L. (2004). Multidimensional assessment of emotion regulation an dysregulation: Development, factor structure, and initial validation of the Difficulties in Emotion Regulation Scale. *Journal of Psychopathology and Behavioral Assessment, 26,* 41-54.

Faul, F., Erdfelder, E., Lang, A.-G., & Buchner, A. (2007). G*Power 3: A flexible statistical power analysis program for the social, behavioral, and biomedical sciences. *Behavior Research Methods,39*, 175-191.

First, M.,B., Gibbon, M., Spitzer, R.L., Williams, J. B.W.. Benjamin, L.S. (1998). *SCID II, Structured Clinical Interview for DSM-IV-Axis II Disorders*. Danderyd: Pilgrim Press.

Friberg, Å. (2006). Känslig för känslor?: En kvantitativ studie av ett känsloregleringsperspektiv på oro och Generaliserat ångestsyndrom. *Unpublished Manuscript*. Stockholm. Department of Psychology. Retrieved from http://www.diva-portal.org/smash/get/diva2:189373/FULLTEXT01.pdf

Guy, W. (1976). The Clinical Global Impression Scale. In: ECDEU Assessment Manual for Psychopharmacology-Revised. Rockville, MD: US Dept. of Health, Education and Welfare, ADAMHA, MIMH Psychopharmacology Research Branch, pp 218-222.

Helleman, M., Goossens, P. J. J., Kaasensbrood, A., & van Achterberg, T. (2014). Experiences of patients with borderline personality disorder with the brief admission intervention: A phenomenological study. *International Journal of Mental Health Nursing.*

Helleman, M., Goossens, P. J. J., Kaaseenbrood, A., & van Achterberg, T. (2013). Evidence base and components of brief admission as an intervention for patients with borderline personality disorder: A review of the literature. *Perspectives in Psychiatric Care, 50,* 65-75, doi: 10.1111/ppc.12023

Holmqvist, M. & Nylander, L. (2013a) “5 frågor”. *Unpublished manuscript*. Lund: Sweden

Holmqvist, M. & Nylander, L. (2013b) Angående screening formuläret “5 frågor”. *Unpublished manuscript*. Lund: Sweden

Kessler, R.C., Adler, L., Ames, M., Demler, O., Faraone, S., Hiripi, E., Howes, M.J., Jin, R., Secnik, K., Spencer, T., Ustun, T.B., Walters, E.E. (2005).The World Health Organization Adult ADHD Self-Report Scale (ASRS): a short screening scale for use in the general population. *Psychological Medicine 35*, 245–256.

Klonsky, E. D., & Glenn, C. R. (2009). Assessing the functions of non-suicidal self-injury: Psychometric properties of the inventory of statements about self-injury (ISAS). *Journal of Psychopathology and Behavioral Assessment, 31*, 215-219.

Koekkoek, B., van der Snoek, R., Oosterwijk, K., van Meijel, B. (2010). Preventive psychiatric admission for patients with borderline personality disorder: a pilot study. *Perspectives in psychiatric care, 46*,127-34

Lecrubier, Y., Sheehan, D., Weiller, E., Amorim, P., Bonora, I., Sheehan, K., Janavs, J. & Dunbar, G. (1997) The M.I.N.I. International Neuropsychiatric Interview (M.I.N.I.) A Short Diagnostic Structured Interview: Reliability and Validity According to the CIDI. *European Psychiatry 12*, 224-231.

Lieb, K., Zanarini, M. C., Schmahl, C., Linehan, M. M. & Bohus, M. (2004). Borderline personality disorder. *The Lancet, 364,* 453-461.

Liljedahl, S. I., Helleman, M., Daukantaitė, D., & Westling, S. (2017). Brief Admission: Manual for training and implementation developed from the Brief Admission Sk.ne Randomized Controlled Trial (BASRCT). Lund, Sweden: Media-Tryck, Lund University.

Liljedahl S, Westling S: A unified theoretical framework for understanding suicidal and self-harming behavior: Synthesis of diverging definitions and perspectives. Presented at the 3rd International Conference on Borderline Personality Disorder and Allied Disorders. Rome, Italy; 2014.

Lindholm, T., Bjärehed, J., & Lundh, L.-G. (2011). Functions of Nonsuicidal Self-Injury among Young Women in Residential Care: A Pilot Study with the Swedish Version of the Inventory of Statements about Self-Injury. Cognitive Behaviour Therapy, 40, 183-189.

Linehan, M. M. (1993). *Cognitive behavioral therapy of borderline personality disorder.* New York, NY: Guilford Press.

Lundh, L-G., (2014). Behandling vid icke-suicidalt självskadebeteende kräver tydlig struktur. Randomiserade kontrollerade studier visar evidens för DBT och MBT. *Läkartidiningen 111:* CH9E

Lundh, L-G., (2013). *Behandlingseffekter på självskadebeteende:Vad visar forskningen?* Retrieved from: http://www.nationellasjalvskadeprojektet.se/download/18.3f4c6c4a13e6133832c137b/1371623590809/Lundh+Forsknings%C3%B6versikt+behandling+sj%C3%A4lvskadebeteende+%282%29.pdf

Nadort, M., Arntz, A., Smit, J.H., Giesen-Bloo, J., Eikelenboom, M., Spirnhoven, P., van Asselt, T., Wensing, M. & van Dyck, R. (2009). Implementation of outpatient schema therapy for borderline personality disorder: study design. *BMC Psychiatry 9,* 64

NICE, (2009). Borderline personality disorder: Treatment and Management. NICE clinical guideline 78. Retrieved from: http://www.nice.org.uk/nicemedia/live/12125/42900/42900.pdf

NICE, (2004). *Self-Harm: the Short-Term Physical and Psychological Management and Secondary Prevention of Self-Harm in Primary and Secondary Care*. NICE clinical guideline 16. Retrieved from: http://publications.nice.org.uk/self-harm-cg16/guidance#issues-for-all-services-and-healthcare-professionals

Odelius, C. & Ramklint, M. (2014). En nationell kartläggnng av förekomsten av självskadande beteende hos patienter inom barn & ungdoms- och vuxenpsykiatrin. Nationella Självskadeprojektet och Uppsala Universitet. Retrieved at http://www.nationellasjalvskadeprojektet.se/download/18.1344ba471455d53fa1b559/1397728643998/Slutrapport+prevalensmätning+140410.pdf

Saunders, J, B,, Aasland, O.G., Babor, T. F., Delafuente, J.R., Grant, M. (1993) Development of the alcohol-use disorders identification test (AUDIT) - WHO collaborative project on early detection of persons with harmful alcohol-consumption-II. *Addiction 88*, 791–804

Sheehan, D.V., Lecrubier, Y., Harnett-Sheehan, K., Amorim, P., Janavs, J., Weiller, E., Hergueta, T., Baker, R.. & Dunbar, G. (1998). The M.I.N.I. International Neuropsychiatric Interview (M.I.N.I.): The Development and Validation of a Structured Diagnostic Psychiatric Interview. *Journal of Clinical Psychiatry 59 (suppl 20):*22-33.

Sheehan, D.V., Lecrubier, Y., Harnett-Sheehan, K., Janavs, J., Weiller, E., Bonara, L.I., Keskiner, A., Schinka, J., Knapp, E., Sheehan, M.F. & Dunbar, G.C. (1997) Reliability and Validity of the M.I.N.I. International Neuropsychiatric Interview (M.I.N.I.): According to the SCID-P. *European Psychiatry 12*, 232-241.

Socialstyrelsen. (2013). *Satisfaction with social welfare services – A review.* Stockholm: Socialstyrelsen.

Socialstyrelsen (2013). Skador och förgiftningar behandlade i slutenvård 2012. Retrieved from: http://www.socialstyrelsen.se/publikationer2013/2013-10-22

Stoffers, J. M., Völlm, B. A., Rücker, G., Timmer A., Huband, N., Lieb, K. (2012). Psychological therapies for people with borderline personality disorder. *The Cochrane database of systematic reviews 15*,8

Støvind, H., Hanneborg, E.M., Ruud, T. (2012). Bedre tid med brukerstyrte inleggelser? *Sykepleien. 100*, 62-4.

World Health Organization (WHO: 2014). *World health organization disability assessment schedule II* (WHODAS 2.0.): Retrieved from: http://www.who.int/classifications/icf/whodasii/en/

Åkerman, S. & Eriksson, T. (2011). *Slutstation rättspsyk: om tvångsvårdade kvinnor som inte dömts för brott.* Stockholm: Natur och Kultur
