# Supplementary material for: Effect of Brief Admission to Hospital by Self-referral for Individuals Who Self-harm and Are at Risk of Suicide: A Randomized Clinical Trial
Source: JAMA Netw Open. 2019 Jun 7;2(6):e195463. doi: 10.1001/jamanetworkopen.2019.5463 (PMC6563573; doi:10.1001/jamanetworkopen.2019.5463)
Supplement: Supplement 2. — eTable 1. Strategies Applied in Brief Admission Skåne Randomized Controlled Trial (BASRCT) for Enhancing Treatment Fidelity eTable 2. Growth Curve Estimates for Total Days Admitted to Hospital eTable 3. ZIP Growth Curve Modeling Estimates for the Count Variables eTable 4. Growth Curve Estimates for the WHODAS Domains eFigure 1. The Number of Days With General Admission to Hospital, With Compulsory Admission to Hospital, and With BA for the BA Intervention Group (A) and for the Control Group (B) Over 3 Time Points eFigure 2. The Mean Scores for the WHODAS Domains at 3 Time Points for the BA Intervention and Control Groups [file jamanetwopen-2-e195463-s002.pdf]

Supplementary Online Content

Westling S, Daukantaitė D, Liljedahl SI, et al. Effect of brief admission to hospital by self-referral for individuals who self-harm and are at risk of suicide: a randomized clinical trial. *JAMA Netw Open*. 2019;2(6):e195463. doi:10.1001/jamanetworkopen.2019.5463

eTable 1. Strategies Applied in Brief Admission Skåne Randomized Controlled Trial (BASRCT) for Enhancing Treatment Fidelity

eTable 2. Growth Curve Estimates for Total Days Admitted to Hospital

eTable 3. ZIP Growth Curve Modeling Estimates for the Count Variables

eTable 4. Growth Curve Estimates for the WHODAS Domains

eFigure 1. The Number of Days With General Admission to Hospital, With Compulsory Admission to Hospital, and With BA for the BA Intervention Group (A) and for the Control Group (B) Over 3 Time Points

eFigure 2. The Mean Scores for the WHODAS Domains at 3 Time Points for the BA Intervention and Control Groups

This supplementary material has been provided by the authors to give readers additional information about their work.

© 2019 Westling S et al. *JAMA Network Open.*

eTable 1. Strategies Applied in Brief Admission Skåne Randomized Controlled Trial (BASRCT) for Enhancing Treatment Fidelity[1]

Fidelity strategy	*Application in BASRCT*
Study design	– A panel of experts consulted before the start of the study, including senior researchers and individuals with lived experience<br>– A fixed maximal dose was decided for the intervention<br>– Established routines for monitoring deviations and giving feedback in case of deviations<br>– Plan for handling implementation setbacks.<br>– Methods for monitoring the BA dose included videotaping of the negotiation process, provider and participant self-report forms, and review of medical records
Provider training	– The training was standardized according to a written manual and adapted for different learning styles.<br>– Three different training sessions were developed and delivered to staff based on previous education and their role in delivering the intervention.<br>– For the providers delivering the core components of the intervention, training included role play, rating of videotaped sessions with feedback during monthly supervisions, and on-demand telephone consultations and coaching 24/7, for situations that must be solved immediately.<br>– During the first three months (pilot phase) of the study, all videotapes were rated for adherence and subsequently 20% were rated.<br>– Supervision and consultation were provided by members of the research team who consulted each other on a weekly basis for questions not explicitly answered by the training manual.<br>– All staff at every ward providing BA was trained to deliver the intervention.<br>– All trainings were delivered by the PI.
Treatment delivery	– The videotapes from the negotiation processes were rated for adherence (see above) and feedback was given to providers on a monthly basis.<br>– Emphasis was put on creating a good relationship between the PI and providers to increase the likelihood of them reporting deviations.<br>– Two standardized questionnaires were developed, for providers and participants to repeatedly, regularly, and independently assess whether the core components of the intervention were delivered.<br>– A protocol for all relevant routines and that describes the preferred provider approach was extracted from the manual and accessible to all providers on the four sites.<br>– To minimize contamination, only one ward at every site received the training and delivered the intervention. Participants in the control group were to the greatest extent possible (with respect to shortage of beds in the region) given hospital admission at wards with no training in the intervention.
Treatment receipt	– Providers were instructed to ask questions and discuss the content of the contract, and the participant was required to write parts of the text during the negotiation process to ensure that the information was understood and to suggest behavior changes emanating from the participant.

© 2019 Westling S et al. *JAMA Network Open.*

	– No participants diagnosed with learning disabilities were included.
Treatment enactment	– BA was evaluated by the participant and provider, face to face as well as confidentially via a computerized form including questions on all the core components of the intervention.<br>– All contracts were evaluated and renegotiated every six months, or before if needed. Providers were instructed to carefully ask and ensure that the participant had understood what was said and specifically ask why the individual had not used BA.

[1]Bellg et al, 2004; Borrelli et al., 2005; Borrelli, 2011

Abbreviations: BASRCT, Brief Admission Randomized Controlled Trial; BA, Brief Admission

© 2019 Westling S et al. *JAMA Network Open.*

eTable 2. Growth Curve Estimates for Total Days Admitted to Hospital

	**Total days admitted to hospital**	
	Intercept	Slope
Group	9.782 (7.50)	-4.058 (4.19)
Intercept	49.279 (5.32)***	-10.634(2.97)***
Residual Variances	1020.388**	389.718**
COV	-152.71 (147.68)	
Chi-square (df = 9)	96.81, p < .001	
CFI	1.000	
TLI	1.065	
SRMR	0.003	
RMSEA	0.000	
90% CI	[0.000; 0.000]	

© 2019 Westling S et al. *JAMA Network Open.*

eTable 3. ZIP Growth Curve Modeling Estimates for the Count Variables

	Visits at the emergency unit		Days with compulsory admission		NSSI	
	Intercept	Slope	Intercept	Slope	Intercept	Slope
	Estimate (SE)	Estimate (SE)	Estimate (SE)	Estimate (SE)	Estimate (SE)	Estimate (SE)
Group	-0.162 (0.205)	0.191 (0.124)	0.884 (0.591)	-0.331 (0.311)	0.576 (0.327)	-0.414 (0.244)
OR (95% CI)	0.850 (-0.803, 0.232)	1.211 (-0.348, 0.956)	2.421 (0.004, 0.215)	0.718 (-0.542, -0.236)	1.779 (-0.976, -0.368)	0.661 (0.168, 1.661)
Mean (ii/si)	-3.559 (0.312)***	1.344 (0.158)***	0.621 (0.184)**	0.109 (0.115)		0.603 (0.268)*
Intercepts (i/s)	1.782 (0.145)***	-0.202 (0.081)*	3.458 (0.530)***	1.217 (0.294)***	2.169 (0.247)***	-0.019 (0.188)

Note: SE = standard error; OR = odds ratio; 95% CI = 95% confidence intervals, *p < .05, **p < .01, ***p < .001.

© 2019 Westling S et al. *JAMA Network Open.*

eTable 4. Growth Curve Estimates for the WHODAS Domains

	D1: cognition		D2: mobility		D3: self-care		D4: getting along		D51: household activities		D6: participation	
	Intercept	Slope	Intercept	Slope	Intercept	Slope	Intercept	Slope	Intercept	Slope	Intercept	Slope
Group	-1.07 (3.82)*	-2.70 (2.11)	1.97 (4.69)	-5.59 (2.34)*	-5.98 (4.19)	0.47 (2.44)	-5.52 (4.77)	1.08 (2.37)	-4.65 (5.43)	1.03 (3.40)	-2.43 (3.46)	-0.59 (1.98)
Intercept	55.30 (2.73)***	2.53 (1.50)	42.24 (3.39)***	-1.57 (1.75)	42.58 (3.05)	-2.31 (1.79)	62.05*** (3.47)	-3.00 (1.77)	68.25 (3.83)***	-4.98 (2.43)	61.82 (2.49)***	-2.02 (1.44)
Variances	217.42*	4.06	211.38	-50.35	206.63*	17.10	401.37**	28.64	426.06*	69.81	22.08	-76.32
COV	-11.36 (55.92)		75.31 (78.92)		44.80 (64.48)		-24.83 (71.91)		3.15 (101.55)		88.95 (52.74)	
Chi-square (df = 9)	50.76, p < .001		56.75, p < .001		66.71, p < .001		73.67, p < .001		61.07, p < .001		39.53, p < .001	
CFI	1.000		1.000		0.958		1.000		0.989		1.000	
TLI	1.006		1.080		0.875		1.047		0.968		1.053	
SRMR	0.028		0.018		0.042		0.017		0.032		0.032	
RMSEA	0.000		0.000		0.104		0.000		0.050		0.000	
90% CI	(0.000, 0.181)		(0.000, 0.131)		(0.000, 0.235)		(0.000, 0.147)		(0.000, 0.197)		(0.000, 0.165)	

Note. 95% CI = 95% confidence intervals; #p < .10, *p < .05, **p < .01, ***p < .001.

© 2019 Westling S et al. *JAMA Network Open.*

eFigure 1. The Number of Days With General Admission to Hospital, With Compulsory Admission to Hospital, and With BA for the BA Intervention Group (A) and for the Control Group (B) Over 3 Time Points

Abbreviation: BA, Brief admission. T1, Time 1; T2, Time 2; T3, Time 3.

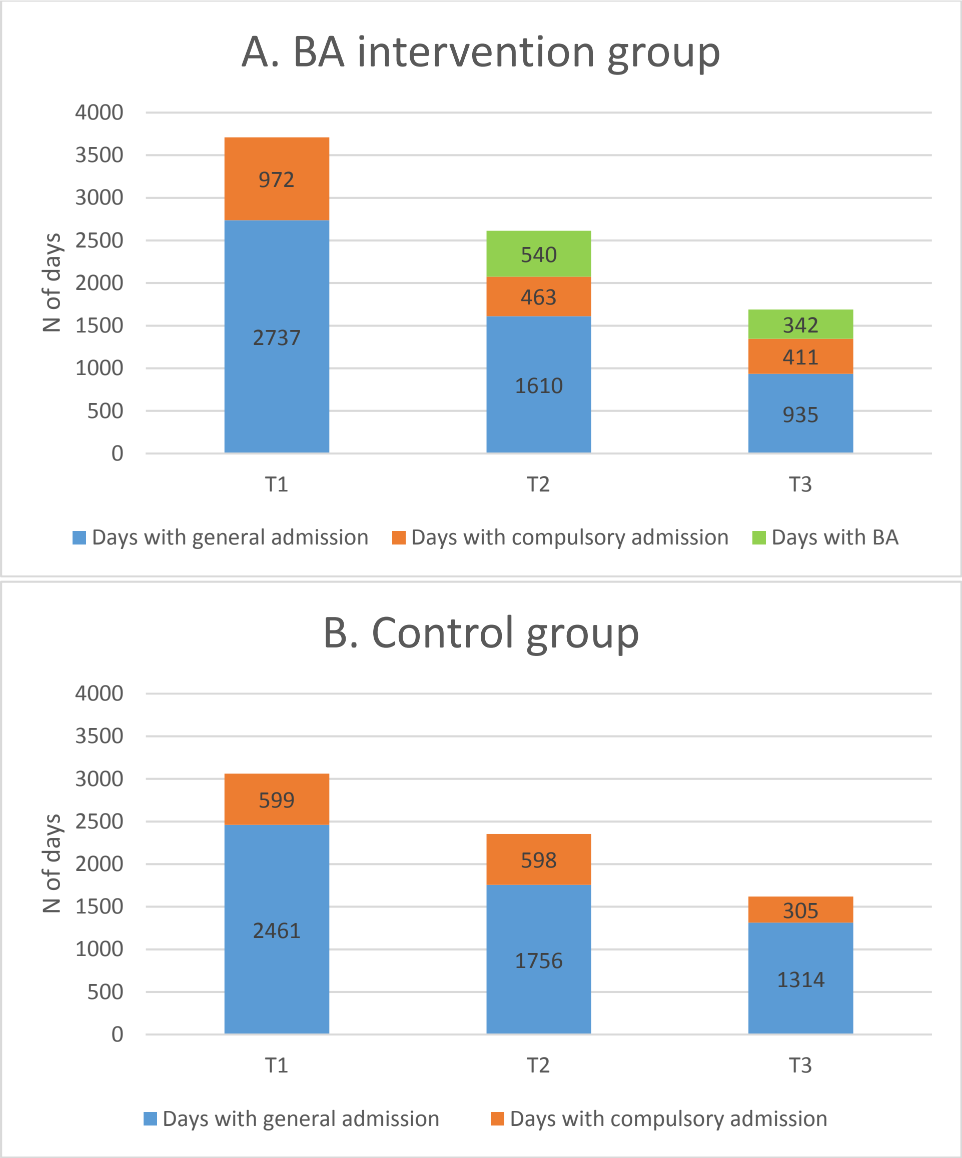

© 2019 Westling S et al. *JAMA Network Open.*

eFigure 2. The Mean Scores for the WHODAS Domains at 3 Time Points for the BA Intervention and Control Groups

Abbreviations: BA, Brief admission; WHODAS, World Health Organization Disability Assessment Schedule

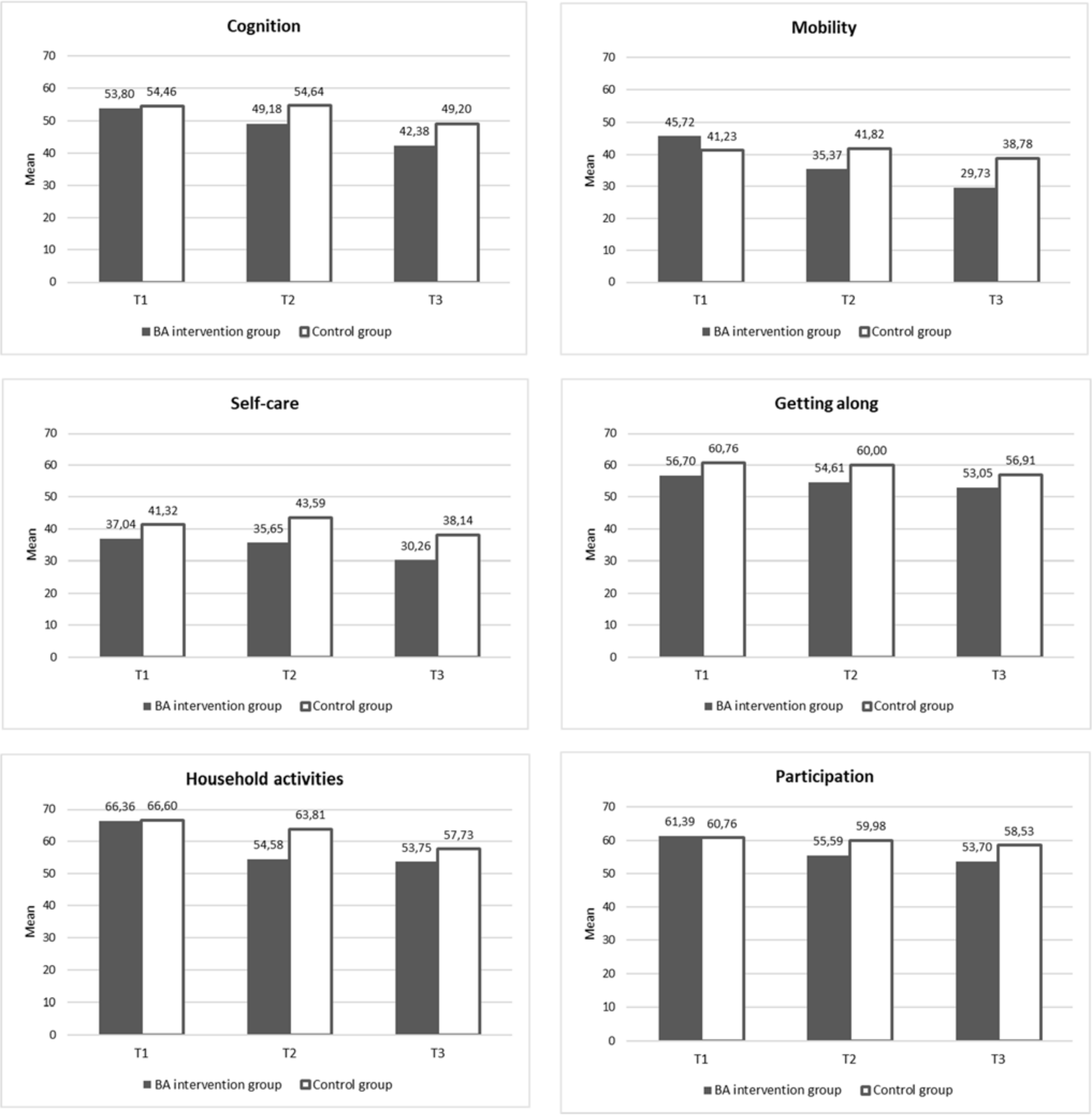

© 2019 Westling S et al. *JAMA Network Open.*
