# Supplementary material for: Effect of Brief Admission to Hospital by Self-referral for Individuals Who Self-harm and Are at Risk of Suicide: A Randomized Clinical Trial
Source: JAMA Netw Open. 2019 Jun 7;2(6):e195463. doi: 10.1001/jamanetworkopen.2019.5463 (PMC6563573; doi:10.1001/jamanetworkopen.2019.5463)
Supplement: Supplement 3. — Data Sharing Statement [file jamanetwopen-2-e195463-s003.pdf]

Data Sharing Statement

Westling. Effect of Brief Admission to Hospital by Self-referral for Individuals Who Self-harm and Are at Risk of Suicide. *JAMA Netw Open*. Published June 07, 2019. 10.1001/jamanetworkopen.2019.5463

Data
Data available: No

Additional Information
Explanation for why data not available: In our approval from the regional ethical review board, it is stated that data should only be presented on a group level as these might contain sensitive information
